# Stereotypic generation of axial tenocytes from bipartite sclerotome domains in zebrafish

**DOI:** 10.1371/journal.pgen.1007775

**Published:** 2018-11-02

**Authors:** Roger C. Ma, Craig T. Jacobs, Priyanka Sharma, Katrinka M. Kocha, Peng Huang

**Affiliations:** Department of Biochemistry and Molecular Biology, Cumming School of Medicine, Alberta Children’s Hospital Research Institute, University of Calgary, Calgary, Canada; Fred Hutchinson Cancer Research Center, UNITED STATES

## Abstract

Development of a functional musculoskeletal system requires coordinated generation of muscles, bones, and tendons. However, how axial tendon cells (tenocytes) are generated during embryo development is still poorly understood. Here, we show that axial tenocytes arise from the sclerotome in zebrafish. In contrast to mouse and chick, the zebrafish sclerotome consists of two separate domains: a ventral domain and a previously undescribed dorsal domain. While dispensable for sclerotome induction, Hedgehog (Hh) signaling is required for the migration and maintenance of sclerotome derived cells. Axial tenocytes are located along the myotendinous junction (MTJ), extending long cellular processes into the intersomitic space. Using time-lapse imaging, we show that both sclerotome domains contribute to tenocytes in a dynamic and stereotypic manner. Tenocytes along a given MTJ always arise from the sclerotome of the adjacent anterior somite. Inhibition of Hh signaling results in loss of tenocytes and enhanced sensitivity to muscle detachment. Together, our work shows that axial tenocytes in zebrafish originate from the sclerotome and are essential for maintaining muscle integrity.

## Author summary

The coordinated generation of bones, muscles and tendons at the correct time and location is critical for the development of a functional musculoskeletal system. Although it is well known that tendon is the connective tissue that attaches muscles to bones, it is still poorly understood how tendon cells, or tenocytes, are generated during embryo development. Using the zebrafish model, we identify trunk tenocytes located along the boundary of muscle segments. Using cell tracing in live animals, we find that tenocytes originate from the sclerotome, an embryonic structure that is previously known to generate the trunk skeleton. In contrast to higher vertebrates, the zebrafish sclerotome consists of two separate domains, a ventral domain and a novel dorsal domain. Both domains give rise to trunk tenocytes in a dynamic and stereotypic manner. Hedgehog (Hh) signaling, an important cell signaling pathway, is not required for sclerotome induction but essential for the generation of sclerotome derived cells. Inhibition of Hh signaling leads to loss of tenocytes and increased sensitivity to muscle detachment. Thus, our work shows that tenocytes develop from the sclerotome and play an important role in maintaining muscle integrity.

## Introduction

Organogenesis relies on the precise and coordinated generation of many different cell types. One such example is the development of the musculoskeletal system, which provides support and mobility to the body. In a functional musculoskeletal system, muscles are attached to bones via tendons, which are essential to transmit mechanical force from muscle to bone. However, despite extensive studies on muscle and skeletal development [1,2], relatively little is known about how tendon cells are generated during development.

In vertebrates, skeletal muscles and the axial skeleton arise from the somites [3–5]. The somite is further subdivided into three compartments as it matures. The sclerotome and dermomyotome are the first to form, and the dermomyotome further splits to form the myotome and the dermatome. Together, these three somitic compartments eventually form much of the trunk, including the skin (dermatome), muscles (myotome), and the axial skeleton (sclerotome).

Hedgehog (Hh) signaling plays an essential role in somite patterning [6–8]. Sonic hedgehog (Shh) is expressed in the notochord and the floor plate, and patterns the surrounding tissues including the somite. In the canonical pathway, Shh functions by binding to its receptor Patched (Ptc), which releases the repression on Smoothened (Smo). Activation of Smo leads to activation of the Gli family of transcription factors, resulting in the expression of downstream target genes, such as the receptor *Ptc*. Mutant analysis and grafting experiments have demonstrated the requirement of Hh signaling in sclerotome development [9]. For example, *Shh* mutant mice have significantly reduced expression of *Pax1*, a sclerotome marker, during early development [10]. Similarly, deletion of both *Gli2* and *Gli3* results in severe loss of sclerotomal marker expression [11]. Conversely, ectopic expression of Shh induces and expands *Pax1* expression [12,13]. Thus, the current model suggests that the canonical Hh pathway via Gli transcriptional activation plays a critical role in sclerotome development.

The sclerotome arises from the ventromedial region of the somite through epithelial-mesenchymal transition (EMT). The formation of the axial skeleton by the sclerotome is largely mediated by the combined action of four transcription factors, Pax1, Pax9, Nkx3.1, and Nkx3.2 (also known as Bapx1) [14–17]. In addition to the axial skeleton, the sclerotome also contributes to the formation of axial tendons [18–20]. Tendons contain fibroblasts (termed tenocytes) embedded in extensive collagen-rich extracellular matrix [21]. Analysis of the expression of *Scleraxis* (*Scx*), encoding a tendon progenitor specific transcription factor, reveals that axial tenocytes are located along the junctions of somite borders in both mouse and chick [19,22]. Although this *Scx*-expressing domain is defined as a fourth somitic compartment termed the syndetome, genetic analysis in chick reveals that the syndetome (and thus axial tenocytes) is induced from *Pax1*^*+*^ sclerotome along the sclerotome-myotome interface [18,19]. However, it remains unknown whether the generation of tendon progenitors from the sclerotome is an evolutionarily conserved process.

Land animals and fish species have very different requirements for bones and muscles. Land animals typically have more sophisticated skeletons to combat gravity and provide support and motility to the body. By contrast, fish can rely on the buoyancy of water to help support their bodies and thus depend less on a robust skeletal system, but they require large blocks of skeletal muscles to help them navigate through their viscous environment. This raises the question of whether the sclerotome has evolved differently to accommodate these different needs in fish. Early somite patterning in zebrafish follows a largely conserved developmental program as in mouse and chick [8]. In both zebrafish and medaka, expression of the sclerotomal markers *pax9* and *twist1* in the ventral region of the somite [23–25] suggests that basic sclerotome development is conserved between higher vertebrates and teleosts. Indeed, cell tracing experiments in zebrafish demonstrate that the presumptive sclerotome contributes to the formation of the vertebral column [7,26]. Morpholino knock-down of key sclerotome genes in medaka, such as *pax1*, *pax9* and *twist1*, causes defects in vertebral column formation [25]. These results suggest a similar role for the sclerotome in skeleton development in teleosts.

In zebrafish, tendon progenitor cells can be identified by the expression of *scleraxis a* (*scxa*), similar to mouse and chick [27]. Although cranial tendon progenitors are shown to be derived from the neural crest lineage [27], it remains unknown how axial tendon progenitors are generated. Zebrafish axial tendon progenitors are located along the somite boundary, a structure known as the myotendinous junction (MTJ). The MTJ is functionally homologous to mammalian tendons, allowing the transmission of force between muscles during fish swimming. The structure of MTJ is maintained by the interaction between the extracellular matrix and the dystrophin-dystroglycan protein complex on the muscle membrane. Defects in MTJ, for instance in *dystrophin* or *dystroglycan* zebrafish mutants, result in severe muscle detachment [28–31], reminiscent of phenotypes in muscular dystrophy patients. However, it is not clear how tendon cells interact with MTJ and regulate muscle attachment.

Here we describe the developmental origins of axial tendon cells in zebrafish. Through marker analysis, we show that the zebrafish sclerotome comprises two separate domains, distinct from mouse and chick. Hh signaling is not required for the induction of sclerotome but instead is essential for the migration and sustained marker expression of sclerotome derived cells. *In vivo* time-lapse imaging reveals that both sclerotome domains contribute to tenocyte formation in a dynamic and stereotypic fashion. Thus, our results suggest that the generation of axial tenocytes from the sclerotome is an evolutionarily conserved process.

## Results

### Characterization of zebrafish sclerotome

**Fig 1:**
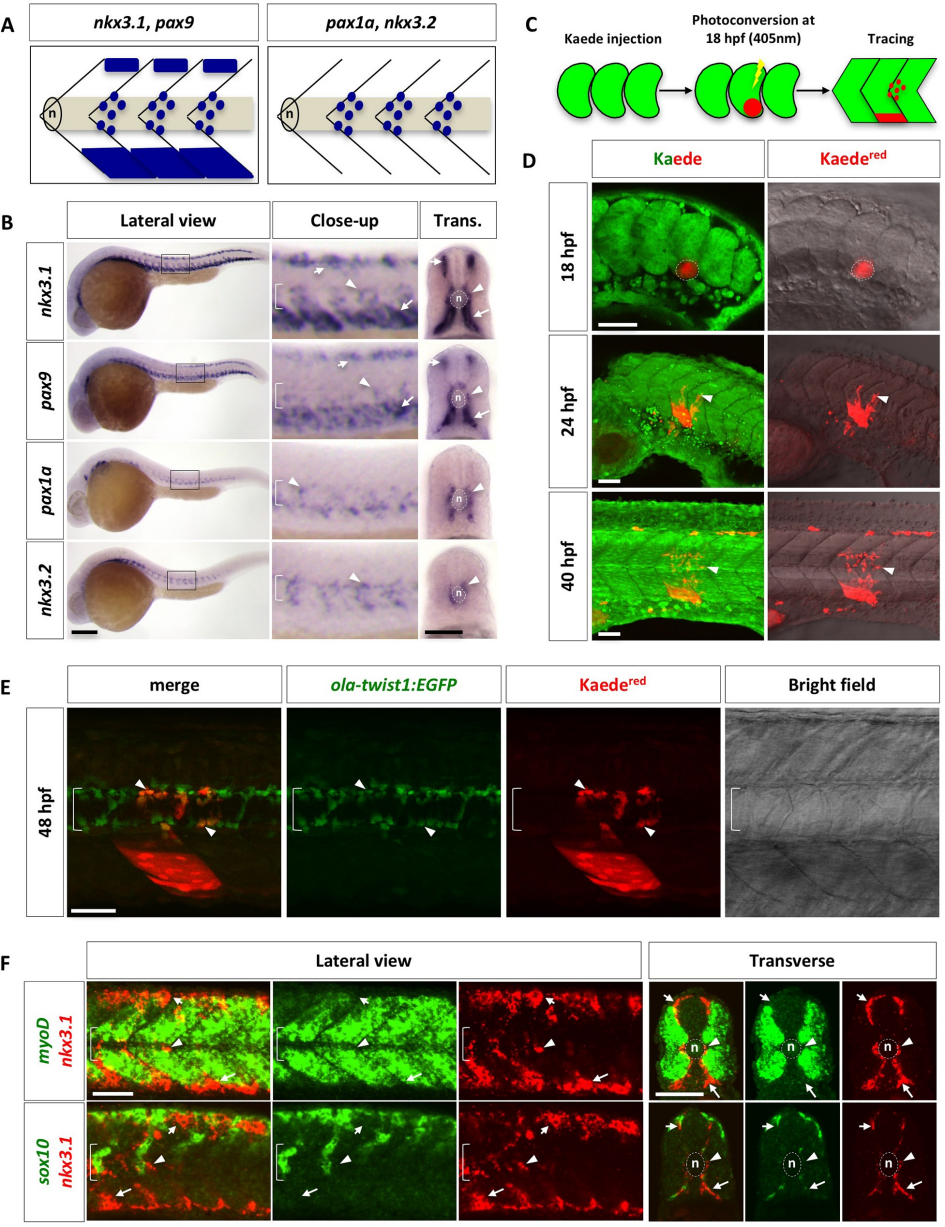
Characterization of the zebrafish sclerotome. (A) Schematic representation of two patterns of sclerotome marker expression. *nkx3*.*1* and *pax9* are expressed in both the dorsal and ventral sclerotome domains along with sclerotome derived notochord associated cells. In contrast, *pax1a* and *nkx3*.*2* are expressed only in sclerotome derived notochord associated cells. Lateral views of three somites are shown with the notochord (n) indicated. (B) Expression of sclerotome markers in wild-type zebrafish at 24 hpf in lateral and transverse (Trans.) views. Close-up images are expanded views of boxed regions in lateral images. *nkx3*.*1* and *pax9* are expressed in three regions: a small domain at the dorsomedial edge of the somite (short arrows), a large domain at the ventromedial region of the somite (long arrows), and cells surrounding the notochord (arrowheads). In contrast, *pax1a* and *nkx3*.*2* are expressed only in cells around the notochord. The notochord (n) is indicated by brackets in close-up views and by dotted lines in transverse views. *n* = 60 embryos per staining. (C) Schematic drawings of Kaede lineage tracing experiment. *Kaede*^*green*^ in the presumptive ventral sclerotome of a single somite at 18 hpf was photoconverted to *Kaede*^*red*^ and followed over time. (D) Lineage tracing of sclerotome derived cells from the ventral sclerotome domain using Kaede. The presumptive ventral sclerotome domain (indicated by dotted line) was photoconverted to *Kaede*^*red*^ at 18 hpf. At 24 hpf, a few sclerotome derived cells (arrowheads) were seen surrounding the notochord. By 40 hpf, many sclerotome derived cells have populated the region surrounding the notochord. *n* = 35 embryos. (E) Lineage tracing of sclerotome derived cells using Kaede in *ola-twist1*:*EGFP* transgenic line. *ola-twist1*:*EGFP* embryos were injected with *Kaede* mRNA at the one-cell stage, and photoconverted in the ventral region of a single somite at 18 hpf as described in (C). At 48 hpf, the red channel was first imaged to identify *Kaede*^*red*^ expressing cells, then the *Kaede*^*green*^ signal was completely photoconverted, and finally, the green channel was imaged in the same z-stack to reveal *ola-twist1*:*EGFP* positive cells. Most *Kaede*^*red*^ sclerotome derived cells surrounding the notochord are positive for *ola-twist1*:*EGFP* (arrowheads). *n* = 21 embryos. (F) Wild-type embryos were co-labeled with the myotome marker *myoD* (green, top panel), or the neural crest marker *sox10* (green, bottom panel) with *nkx3*.*1* (red). *nkx3*.1-expressing dorsal sclerotome (short arrows), ventral sclerotome (long arrows), and sclerotome derived notochord associated cells (arrowheads) do not express either *myoD* or *sox10*. Images shown are lateral and transverse views of embryo at 24 hpf. *n* = 30 embryos per staining. Scale bars: (B) 200 μm in lateral views, 50 μm in transverse views; (D, E, F) 50 μm.

During development, the maturing somite is split to form the dermomyotome, myotome, and sclerotome. To characterize sclerotome development in zebrafish, we determined the expression pattern of key sclerotome transcription factors, which are essential for sclerotome development in higher vertebrates. At 24 hours post fertilization (hpf), two distinct expression patterns were observed by RNA in situ hybridization (Fig 1A and 1B). *nkx3*.*1* and *pax9* were primarily expressed in a domain located at the ventromedial edge of the somite, a smaller domain at the dorsomedial edge of the somite, and a population of cells surrounding the notochord in a metameric pattern. By contrast, *pax1a* and *nkx3*.*2* were only expressed in the cells surrounding the notochord in a repeated pattern along the anteroposterior axis. Double fluorescent in situ hybridization confirmed that the dorsal and ventral sclerotome domains co-expressed *nkx3*.*1* and *pax9*, while cells around the notochord expressed all four markers (S1A Fig). Analysis of additional sclerotome markers, including *twist1b*, *versican b*, and *foxc1b*, revealed similar expression patterns as *nkx3*.*1* and *pax9* at 24 hpf (S1B Fig). The marker expression analysis suggests that the zebrafish sclerotome consists of three compartments.

Based on the expression pattern of these sclerotome markers, we hypothesized that the sclerotome gives rise to the cells surrounding the notochord. To test this possibility, we first performed a time course analysis of sclerotome markers (S1C Fig). At 16 hpf, only *nkx3*.*1* was expressed in the ventral regions of somites. At 18 hpf, *nkx3*.*1* mRNA was also detected in the dorsal domains, along with the emergence of *pax9* expression in the ventral domains. By 20 hpf, *nkx3*.*1/pax9* cells appeared to “sprout” from the ventral domain of each somite to surround the notochord in an anterior-to-posterior progression, coinciding with the expression of *pax1a* and *nkx3*.*2* around the notochord. These results suggest that the ventral sclerotome domain likely gives rise to cells surrounding the notochord.

To confirm that cells migrate out of the ventral sclerotome to populate around the notochord, we performed cell tracing experiments using the photoconvertible fluorescent protein, Kaede [32] (Fig 1C and 1D). Wild-type embryos were injected with *Kaede* mRNA at the one-cell stage. At 18 hpf, a small ventral region in a newly formed somite, containing the presumptive ventral sclerotome, was photoconverted to switch all green-fluorescent Kaede protein to red-fluorescent Kaede protein (*Kaede*^*red*^). This targeted photoconversion approach allowed us to strongly label the ventral somite with *Kaede*^*red*^ expression but minimally in deeper tissues (S2A and S2B Fig). The perdurance of *Kaede*^*red*^ protein can be used to trace the dynamics of converted cells over 2 days (Fig 1C). By 24 hpf, a few mesenchymal-like *Kaede*^*red*^ cells were seen to exit from the posterior end of the somite and migrate towards the notochord (Figs 1D and S2C). By 40 hpf, 10–15 cells derived from the ventral sclerotome can be identified surrounding the notochord (Figs 1D and S2C). Interestingly, these *Kaede*^*red*^ cells derived from the presumptive ventral sclerotome always occupied a region about one half-somite more posterior to the somite of origin. Since the photoconversion approach also labeled a small patch of skin cells and weakly some deeper tissues (S2A and S2B Fig), we carried out additional experiments to confirm the sclerotomal identity of *Kaede*^*red*^ cells around the notochord. We performed similar cell tracing experiments in *Kaede* mRNA injected *ola-twist1*:*EGFP* embryos. The medaka (*Oryzias latipes*) *ola-twist1* promoter has previously been shown to drive expression in sclerotome derived cells around the notochord in both medaka and zebrafish [25,33–35]. Indeed, most *Kaede*^*red*^ cells surrounding the notochord were also positive for *ola-twist1*:*EGFP* (Fig 1E). Together, our results suggest that sclerotome derived cells around the notochord originate from the ventral sclerotome.

In mouse and chick, the sclerotome is defined as one single domain located in the ventromedial region of the somite [36,37]. By contrast, we identified a separate dorsal domain expressing several known sclerotome markers in zebrafish (Figs 1B and S1B). To rule out the possibility that the dorsal domain corresponded to non-sclerotome dorsal structures, we performed double fluorescent in situ hybridization using markers labeling the myotome and neural crest cells (Figs 1F, S3A and S3B). At 24 hpf, no co-expression of *nkx3*.*1* with either the myotome marker *myoD*, or the neural crest markers *sox10*, *crestin*, and *mitfa*, was observed in lateral or cross section analysis of zebrafish somites. In addition, *nkx3*.*1*^*+*^ cells around the notochord were distinct from muscle pioneers, labeled by *eng2* expression (S3C Fig). Together, our results reveal that the zebrafish sclerotome initially consists of two separate domains: a ventral domain analogous to that in higher vertebrates and a novel dorsal domain. As the somite matures, the ventral sclerotome domain also contributes to cells around the notochord. We referred to this population of cells as “sclerotome derived notochord associated cells” in this manuscript.

### Distinct roles of Hh signaling in sclerotome development

**Fig 2:**
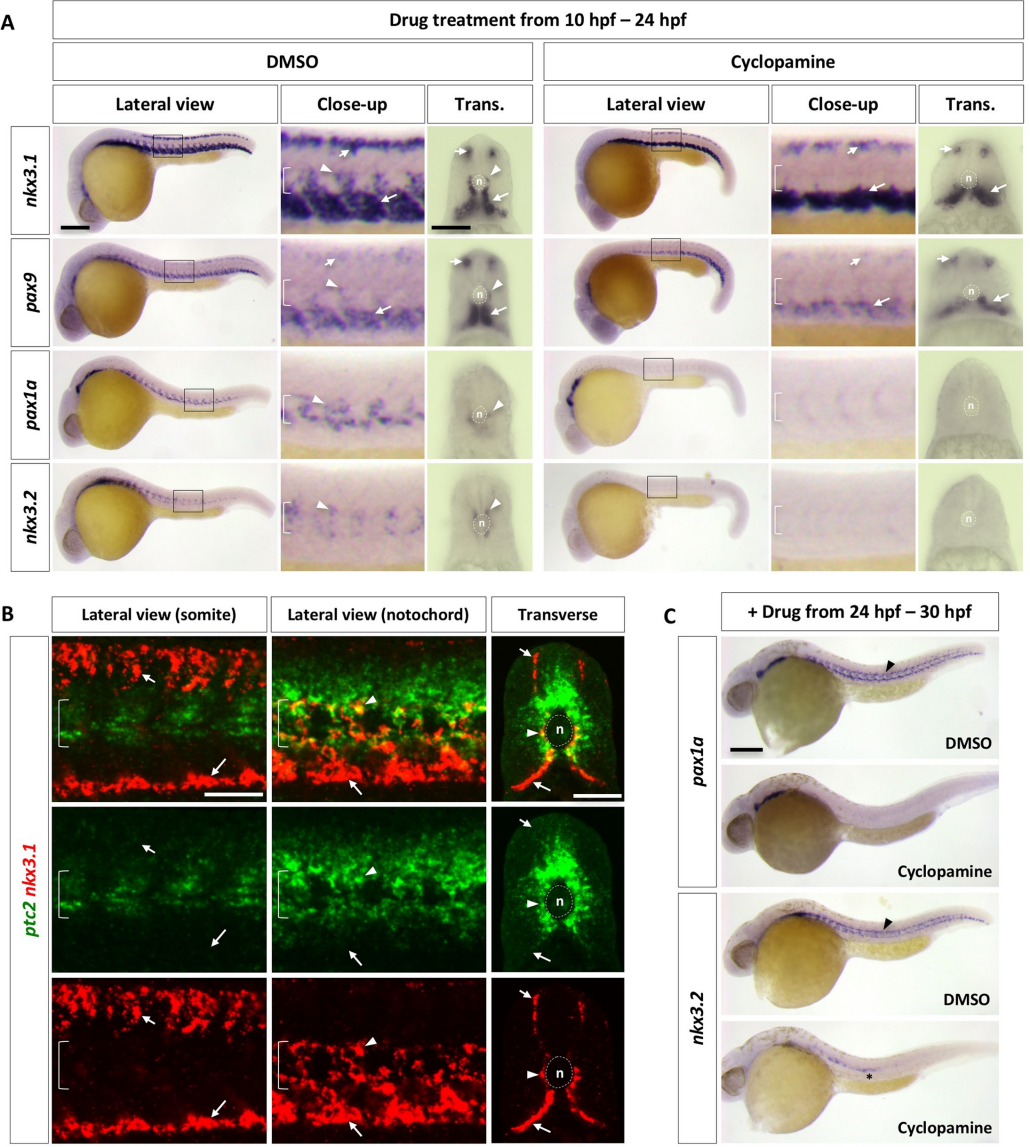
Regulation of the zebrafish sclerotome by Hh signaling. (A) Wild-type embryos were treated with either DMSO or cyclopamine between 10 hpf and 24 hpf, and stained for the expression of *nkx3*.*1*, *pax9*, *pax1a*, and *nkx3*.*2*. The expression of *nkx3*.*1* and *pax9* in dorsal and ventral sclerotome domains (short and long arrows, respectively) remained the same upon cyclopamine treatment, while expression of all sclerotome markers was absent in sclerotome derived notochord associated cells (arrowheads). The notochord (n) is indicated by brackets in lateral views and dotted lines in transverse views. *n* = 45 embryos per condition. (B) Wild-type embryos at 24 hpf were co-labeled with *ptc2* (green) and *nkx3*.*1* (red). *nkx3*.*1* expressing dorsal and ventral sclerotome domains (short and long arrows, respectively) do not express *ptc2*, whereas *nkx3*.*1* expressing cells surrounding the notochord (arrowheads) are positive for *ptc2*. The confocal plane of the somite shows dorsal and ventral sclerotome domains, while the optical slice near the notochord reveals notochord associated cells and the ventral sclerotome. *n* = 15 embryos. (C) Wild-type embryos were treated with either DMSO or cyclopamine between 24 hpf and 30 hpf, and stained for the expression of *pax1a* and *nkx3*.*2* at 30 hpf. *pax1a* and *nkx3*.*2* expression was absent in sclerotome derived notochord associated cells (arrowheads) upon treatment of cyclopamine. Note that *nkx3*.*2* expression in migrating lateral line cells (asterisk) was not affected. *n* = 30 embryos per staining. Scale bars: (A, C) 200 μm in lateral views, 50 μm in transverse views; (B) 50 μm.

Since Hh signaling is essential for sclerotome formation in mouse and chick [10–13], we asked how Hh signaling regulates the development of zebrafish sclerotome. We treated wild-type embryos at the onset of somite formation (10 hpf) with cyclopamine, a specific inhibitor of the Hh signaling pathway [38], and analyzed sclerotome marker expression at 24 hpf (Fig 2A). Expression of *nkx3*.*1* and *pax9* in both the dorsal and ventral sclerotome domains was largely intact in cyclopamine-treated embryos, suggesting that sclerotome can be induced in the absence of Hh signaling. By contrast, expression of *nkx3*.*1*, *pax9*, *pax1a*, and *nkx3*.*2* in sclerotome derived notochord associated cells was completely abolished. To further test the requirement of Hh signaling in sclerotome development, we analyzed a ciliary mutant, *iguana*, which shows loss of the highest level of Hh response, but expansion of low level of Hh pathway activation [39–44]. Similar to those in cyclopamine-treated embryos, both dorsal and ventral sclerotome domains formed normally in *iguana* mutants, but marker expression in sclerotome derived notochord associated cells was largely absent (S4 Fig). Consistent with these results, Hh signaling activity, as indicated by *ptc2* expression, was present in *nkx3*.*1*^*+*^ cells surrounding the notochord but absent in dorsal and ventral sclerotome domains in wild-type embryos at 24 hpf (Fig 2B). Similar results were observed using a sensitive Hh signaling reporter, *ptc2*:*Kaede* [45] (S5A Fig). Photoconversion experiments using the *ptc2*:*Kaede* reporter revealed that cells surrounding the notochord, likely derived from the sclerotome, displayed active Hh response at both 30 hpf and 48 hpf (S5B Fig). Collectively, our results suggest that the initial formation of sclerotome domains is not dependent on Hh signaling, but sclerotome derived notochord associated cells require high levels of Hh signaling.

The absence of marker expression in sclerotome derived notochord associated cells in cyclopamine-treated embryos suggests two non-mutually exclusive possibilities. First, marker expression in these cells is maintained by active Hh signaling. Second, the migration of these cells requires active Hh signaling. To test the first scenario, we blocked Hh signaling at 24 hpf, after cells from the ventral sclerotome have migrated and surrounded the notochord (Fig 2C). Control embryos showed strong *pax1a* and *nkx3*.*2* expression in cells around the notochord at 30 hpf. However, in embryos treated with cyclopamine, *pax1a* and *nkx3*.*2* expression was completely absent around the notochord. This result suggests that sclerotome derived notochord associated cells require active Hh signaling to maintain the expression of sclerotomal markers.

**Fig 3:**
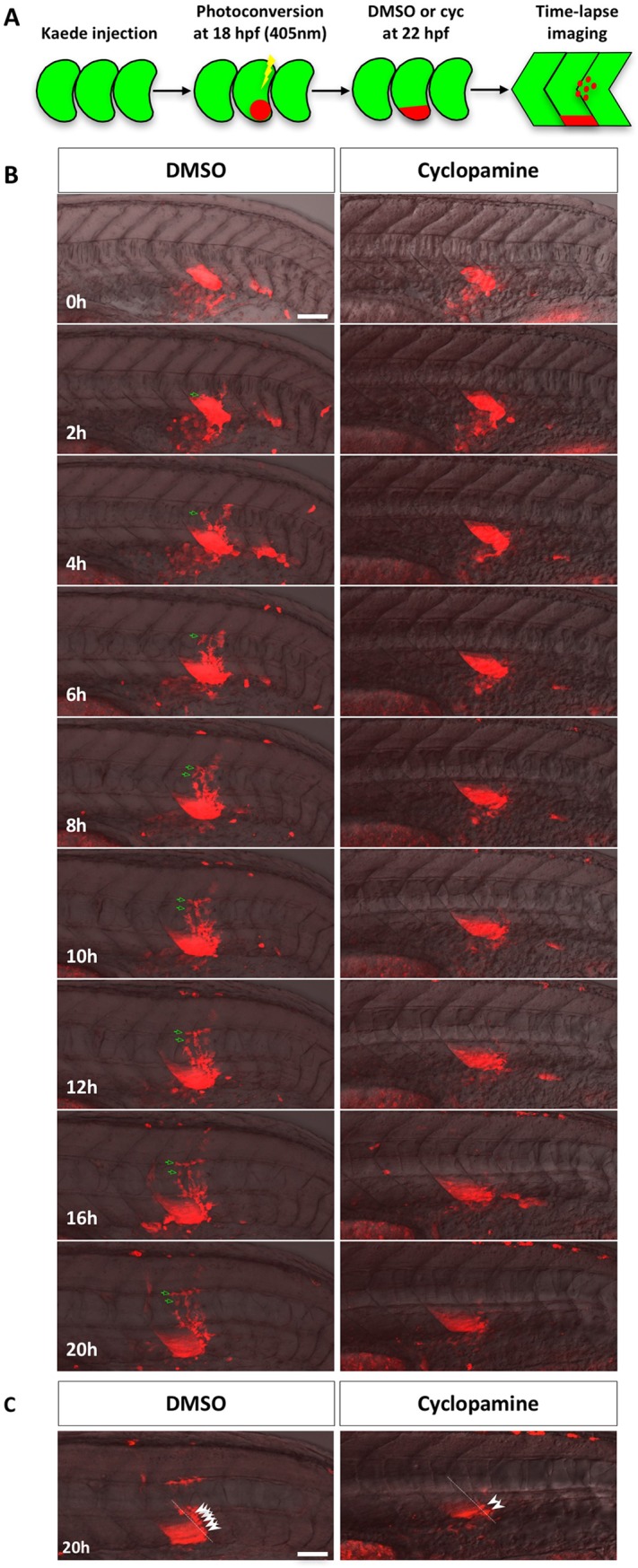
Lineage tracing of sclerotome derived cells. (A) Schematic of the experimental procedure. Embryos were injected with *Kaede* mRNA at the one-cell stage. At 18 hpf, *Kaede*^*green*^ in the presumptive ventral sclerotome of a newly formed somite (somite 17 or 18) was photoconverted to *Kaede*^*red*^. At 22 hpf, photoconverted embryos were treated with either DMSO or cyclopamine and imaged over the next 20 hours. (B) Representative snapshots of different time-points during lineage tracing of sclerotome derived cells between 22 hpf (0h) and 42 hpf (20h). Merged images containing the red channel and the bright field are shown. *Kaede*^*red*^ sclerotome derived cells in DMSO treated controls migrated from the ventral sclerotome to the notochord before dividing to generate a population of cells surrounding the notochord. Green arrows indicate one representative sclerotome derived cell and its progeny. In the presence of cyclopamine, sclerotome derived cells failed to migrate out of the ventral sclerotome to the notochord. The corresponding movies are shown in S1 and S2 Videos. *n* = 7 embryos per condition. (C) Projection of deep confocal slices at the last time point of time-lapse. A population of *Kaede*^*red*^ cells reminiscent of tenocytes (arrowheads) located along the myotendinous junction between somites (dotted lines). Fewer tenocyte-like cells were found in cyclopamine treated embryos. Scale bars: 50 μm.

To determine whether Hh signaling also regulates the cell migration from the ventral sclerotome, we combined cyclopamine with *Kaede*-mediated cell tracing. Similar to previous experiments (Fig 1C), we labeled the presumptive ventral sclerotome domain at 18 hpf by photoconversion, incubated embryos with DMSO or cyclopamine at 22 hpf, and followed *Kaede*^*red*^ sclerotome derived cells from 22 to 42 hpf using time-lapse imaging (Fig 3A). In DMSO-treated embryos, sclerotome derived cells migrate dorsally out from the posterior side of the ventral somite to surround the notochord slightly posterior to the original somite. These cells then divided 1–3 times to generate a population of cells surrounding the notochord (Fig 3B and S1 Video). However, in the presence of cyclopamine, cells failed to migrate out of the ventral sclerotome domain to surround the notochord (Fig 3B and S2 Video). These results suggest that migration of cells from the ventral sclerotome to surround the notochord also requires active Hh signaling. Therefore, although dispensable for the induction of sclerotome domains, Hh signaling is required in multiple steps during sclerotome development: first the migration of sclerotome derived cells, and then the maintenance of marker expression in these cells.

### Zebrafish trunk tenocytes display a unique morphology

**Fig 4:**
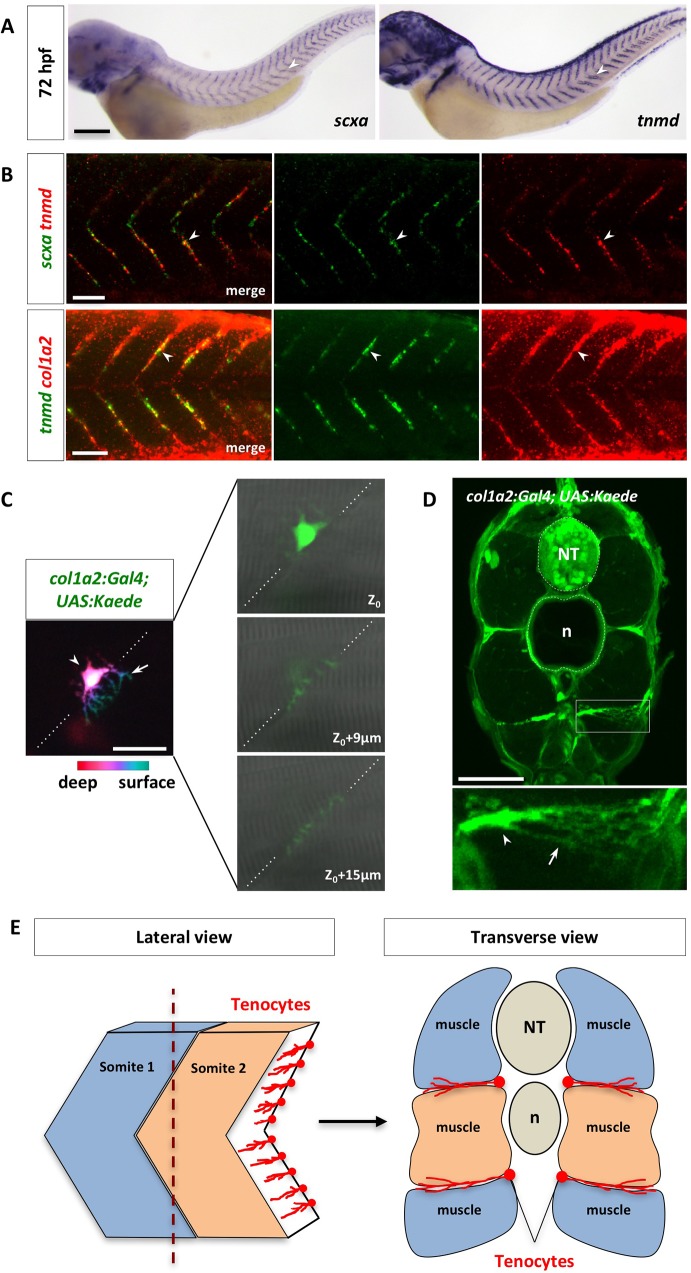
Characterization of zebrafish axial tenocytes. (A) Expression of tenocyte markers *scxa* and *tnmd* in wild-type embryos at 72 hpf. Both *scxa* and *tnmd* are expressed along the V-shaped myotendinous junction (MTJ) between somites (arrowheads). The complete time course of expression is shown in S6 Fig. *n* = 30 embryos per staining. (B) Wild-type embryos at 72 hpf were co-labeled with *scxa* and *tnmd* (green and red in the top panel, respectively), or *tnmd* and *col1a2* (green and red in the bottom panel, respectively). Overlapping expression between *scxa*, *tnmd*, and *col1a2* were observed in tenocytes along the MTJ (arrowheads). *n* = 60 embryos per staining. (C) Mosaic *col1a2*^*Kaede*^ embryos at 3 dpf were selected to image individual *Kaede*^*+*^ tenocytes. Color coded depth projection of a single *col1a2*^*Kaede*^ tenocyte is shown on the left. The tenocyte cell body (arrowhead) is located deeper (magenta) along the MTJ (dotted lines), whereas its cellular processes (arrow) are projected towards the surface (cyan). Three individual confocal slices of the same tenocyte are shown on the right. The cell body is found near the medial edge of the somite (Z_0_) and its cellular projections extend through the MTJ (Z_0_ + 9 μm and Z_0_ + 15 μm) towards the lateral surface. *n* = 25 embryos. (D) Cross section view of *col1a2*^*Kaede*^ embryos at 5 dpf. An expanded view of a tenocyte in a boxed region is shown at the bottom. The cell bodies of tenocytes (arrowhead) are located at the medial edge of the MTJ, and they extend long cellular projections (arrow) towards the surface of the embryo. *n* = 28 tenocytes from 15 embryos. (E) Schematic representations of lateral and transverse views of tenocyte organization along the somite boundaries. NT: neural tube; n: notochord. Scale bars: (A) 200 μm; (B) 50 μm; (C) 20 μm; (D) 50 μm.

Previous work in chick suggests that axial tendon progenitors arise from a sub-compartment of the sclerotome [19,20]. Consistent with this notion, close examination of cell tracing experiments revealed that a population of sclerotome derived *Kaede*^*red*^ cells eventually located along the myotendinous junction (MTJ) between neighboring somites at 42 hpf (Fig 3C), reminiscent of tenocytes in mouse and chick. Zebrafish axial tenocytes can be labeled by the expression of the tenocyte progenitor marker *scleraxis a* (*scxa*), and the differentiated tenocyte makers *tenomodulin* (*tnmd*) and *collagen 1a2* (*col1a2*) [27]. Tenocyte progenitors marked by *scxa* first appeared at 36 hpf, while mature tenocytes labeled by *tnmd* began forming at 42 hpf during development (S6 Fig). Cell bodies of tenocytes were located at the somite boundaries as a single column along each MTJ. By 72 hpf, tenocytes labeled the entire “V” of the MTJ and co-expressed *scxa*, *tnmd*, and *col1a2* (Fig 4A and 4B). To determine the morphology of tenocytes, we generated a *col1a2*:*Gal4; UAS*:*Kaede* transgenic line (referred to as *col1a2*^*Kaede*^) by BAC (bacterial artificial chromosome) transgenesis to label all fibroblasts, including differentiated tenocytes (details of this transgenic line will be described in a separate manuscript). Taking advantage of the mosaic nature of this transgenic line, we were able to visualize the morphology of individual tenocytes. The cell bodies of tenocytes were positioned at the medial edge of the MTJ, and they extended long, tree-like cellular processes within the intersomitic space towards the lateral surface (Fig 4C). This morphology can be better visualized in transverse sections, in which tenocytes extended multiple processes mediolaterally with an average length of 40 μm (Figs 4D and S7). The MTJ, analogous to mammalian tendons, is known to be the major site of muscle attachment in zebrafish [46–48]. The anatomical location and the unique morphology of tenocytes (Fig 4E) suggest that tenocytes play an important role in maintaining muscle attachment.

### The sclerotome gives rise to axial tenocytes

**Fig 5:**
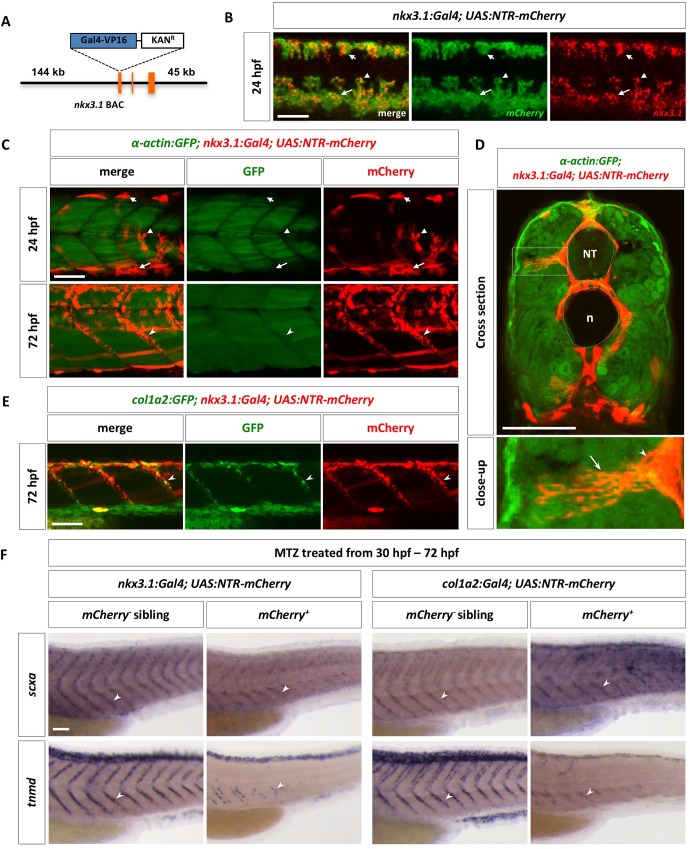
The sclerotome gives rise to axial tenocytes. (A) Schematic representation of the *nkx3*.*1*:*Gal4* BAC reporter. A cassette containing Gal4-VP16 and Kanamycin resistance gene was recombined to replace the first coding exon of *nkx3*.*1*. (B) *nkx3*.*1*^*NTR-mCherry*^ embryos were co-labeled with *nkx3*.*1* (red) and *ntr-mCherry* (green) at 24 hpf. *nkx3*.*1* and *mCherry* show overlapping expression in the dorsal sclerotome (short arrows), the ventral sclerotome (long arrows), and sclerotome derived notochord associated cells (arrowheads). *n* = 30 embryos. (C) The *nkx3*.*1*^*NTR-mCherry*^ line (red) was crossed with the *α-actin*:*GFP* line (green) to label muscle cells. At 24 hpf, most *mCherry*^*+*^ cells, including dorsal sclerotome (short arrows), ventral sclerotome (long arrows), and sclerotome derived notochord associated cells (arrowheads), are not *α-actin*:*GFP* positive. Note that a few elongated *mCherry*^*+*^ muscle fibers are present in dorsal and ventral region of the somite. By 72 hpf, tenocytes (notched arrowheads) are found along the MTJ between adjacent somites. *n* = 5 embryos. (D) Cross section views of *α-actin*:*GFP; nkx3*.*1*^*NTR-mCherry*^ embryos at 72 hpf show tenocytes (arrowheads) near at the medial edge of the somite extending long cellular projections (arrows) towards the surface of the body. The neural tube (NT) and notochord (n) are indicated by the dotted lines. An expanded view of an individual tenocyte in a boxed region is shown. *n* = 3 embryos. (E) Co-labeling of *nkx3*.*1*^*NTR-mCherry*^ (red) with the *col1a2*:*GFP* (green) transgenic reporter at 72 hpf. *mCherry*^*+*^ tenocytes (notched arrowheads) along the MTJ also co-express *col1a2*:*GFP*. *n* = 15 embryos. (F) Embryos from *nkx3*.*1*^*NTR-mCherry*^ or *col1a2*^*NTR-mCherry*^ outcrosses were treated with metronidazole (MTZ) from 30 hpf to 72 hpf, and stained for the expression of *scxa* or *tnmd*. *mCherry*^*+*^ embryos showed markedly reduced numbers of tenocytes (notched arrowheads) as indicated by *scxa* or *tnmd* staining, compared to *mCherry*^*-*^ sibling controls. *n* = 40 embryos per condition. Scale bars: 50 μm.

To directly test whether different sclerotome domains give rise to axial tenocytes, we generated a *nkx3*.*1*:*Gal4* BAC transgenic line to trace the lineage of sclerotome cells. The cDNA encoding the Gal4-VP16 cassette was engineered into a BAC containing the *nkx3*.*1* genomic region, including 144 kb upstream and 45 kb downstream regulatory sequences (Fig 5A). We crossed this *nkx3*.*1*:*Gal4* line with a *UAS*:*Nitroreductase-mCherry* (*UAS*:*NTR-mCherry*) line [49] to label not only the initial sclerotome domains, but also sclerotome derived cells due to the perdurance of the mCherry protein. The *nkx3*.*1*:*Gal4; UAS*:*NTR-mCherry* transgenic line (referred to as *nkx3*.*1*^*NTR-mCherry*^) faithfully recapitulated endogenous *nkx3*.*1* expression, labeling the dorsal sclerotome, ventral sclerotome as well as sclerotome derived notochord associated cells at 24 hpf (Fig 5B). Consistent with our previous observations (Fig 1F), the dorsal and ventral sclerotomes marked by *nkx3*.*1*^*NTR-mCherry*^ appeared to flank the myotome, labeled by *α-actin*:*GFP*, at 24 hpf (Fig 5C). By 72 hpf, some *nkx3*.*1*^*NTR-mCherry*^ positive cells were observed along the MTJ, displaying extensive long cellular projections (Fig 5C and 5D and S3 Video), reminiscent of *col1a2*^*Kaede*^ tenocytes (Fig 4C and 4D). Indeed, *nkx3*.*1*^*NTR-mCherry*^ positive cells along the MTJs co-expressed the *col1a2*:*GFP* transgene (Fig 5E), suggesting that the sclerotome gives rise to tenocytes. To establish the lineage relationship between *nkx3*.*1*^*+*^ sclerotome cells and *col1a2*^*+*^ tenocytes, we performed time-lapse imaging in *nkx3*.*1*^*NTR-mCherry*^*; col1a2*:*GFP* embryos. We found that *mCherry*^*+*^ sclerotome derived cells were weakly positive for *col1a2*:*GFP* at 26 hpf, but as *mCherry*^*+*^ tenocytes emerged at the myotendinous junction, they showed much stronger *col1a2*:*GFP* expression (S4 Video). To further confirm this result, we took advantage of the nitroreductase enzyme expressed in the *UAS*:*NTR-mCherry* reporter which allowed us to ablate NTR-expressing cells. NTR converts the non-toxic prodrug metronidazole (MTZ) into a cytotoxic compound [50,51], thereby allowing us to ablate *nkx3*.*1* specific cells. MTZ treatment of *nkx3*.*1*^*NTR-mCherry*^ embryos from 30 to 72 hpf resulted in a significant reduction of both tenocyte markers, *scxa* and *tnmd* (Fig 5F). Similar results were obtained when *col1a2*^*+*^ cells were ablated using the *col1a2*:*Gal4; UAS*:*NTR-mCherry* transgenic line (Fig 5F). Together, these results suggest that axial tenocytes are derived from the sclerotome lineage during development.

### Stereotypic generation of tenocytes from different sclerotome domains

**Fig 6:**
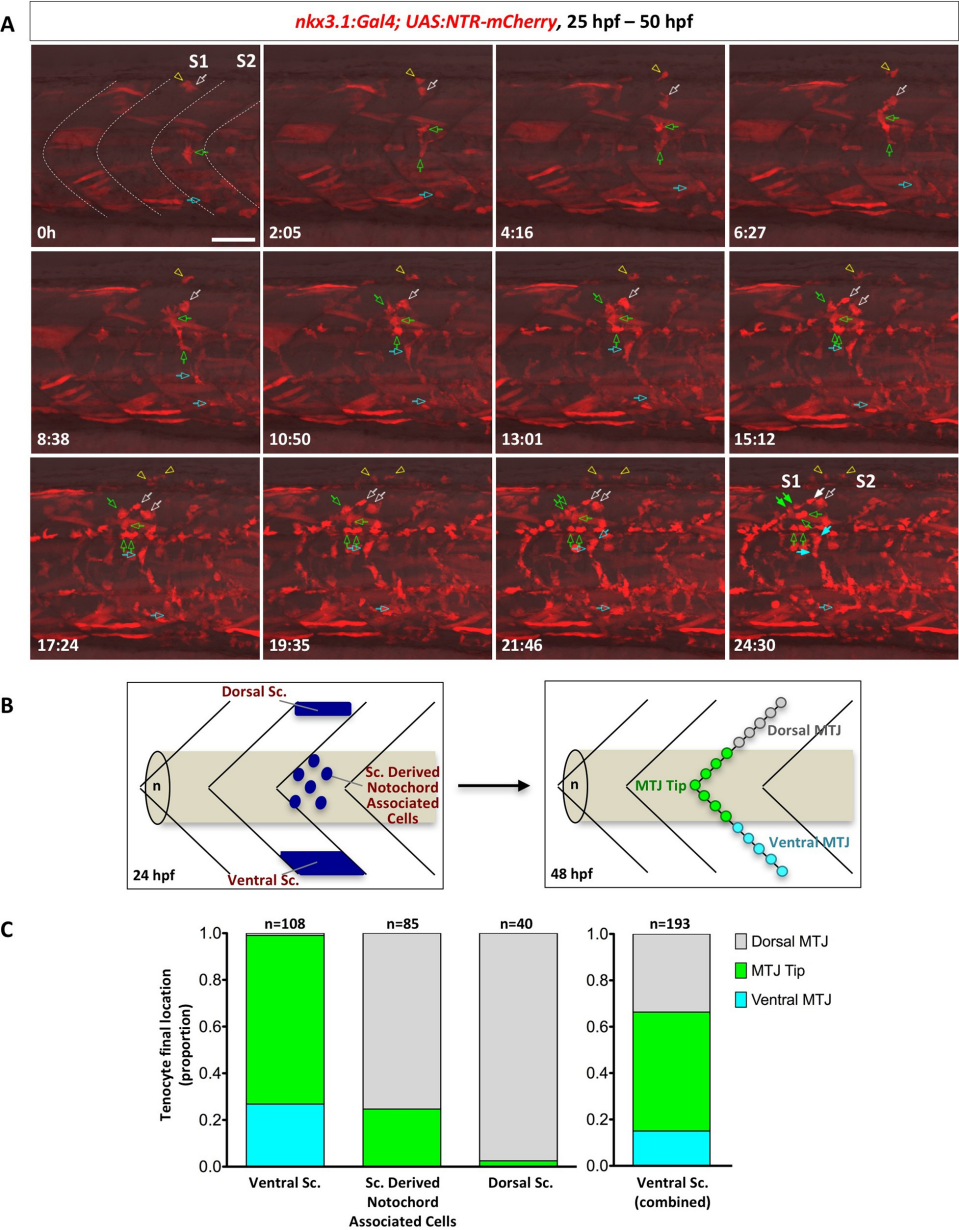
Stereotypic generation of axial tenocytes from different sclerotome domains. (A) Snapshots of time-lapse imaging of an *nkx3*.*1*^*NTR-mCherry*^ embryo between 25 hpf and 50 hpf. Tenocytes were retrospectively traced to determine their cells of origin. Four representative cells (at 0h) from the dorsal sclerotome (2 cells, white arrow and yellow arrowhead), sclerotome derived notochord associated cells (1 cell, green arrow), and the ventral sclerotome (1 cell, cyan arrow) were traced over 25 hours with their daughter cells indicated by the same colored arrows/arrowheads. Based on its initial position, the traced sclerotome derived notochord associated cell was likely derived from the same somite (S1) as the traced dorsal sclerotome cells, whereas the ventral sclerotome cell was from the adjacent posterior somite (S2). Three of the traced cells generated tenocytes along the MTJ (solid arrows) along with several other interstitial cells (open arrows) at the 24.5 hour time frame, whereas one traced dorsal sclerotome cell gave rise to 2 fin mesenchymal cells. The corresponding time-lapse movie is shown in S5 Video. *n* = 7 embryos. (B) Schematic representation of how tenocytes were quantified based on their origin and positioning along the MTJ. Starting locations of each sclerotome cell at 24 hpf were divided into three compartments: the dorsal sclerotome, the ventral sclerotome, or sclerotome derived notochord associated cells. At 48 hpf, tenocytes were quantified based on their final locations along the “V” of each MTJ: dorsal MTJ (grey, above the notochord), ventral MTJ (cyan, below the notochord), or MTJ tip (green, near the notochord). (C) Quantification of contribution of different sclerotome compartments to axial tenocytes. Tenocytes originated from the ventral sclerotome, sclerotome derived notochord associated cells, or the dorsal sclerotome were graphed based on their final locations along the MTJ. Since sclerotome derived notochord associated cells are derived from the ventral sclerotome, the graph on the right depicts the combined contribution of all cells from the ventral domain. Overall, the dorsal sclerotome mainly gives rise to tenocytes found in the dorsal MTJ, while the ventral sclerotome generates tenocytes along the entire MTJ axis. *n* = 233 tenocytes from 7 movies. Scale bars: 50 μm.

The zebrafish sclerotome consists of three compartments at 24 hpf: the dorsal sclerotome domain, the ventral sclerotome domain, and sclerotome derived notochord associated cells originating from the ventral sclerotome (Fig 1A). We next asked how cells from each of these compartments contribute to tenocyte formation. We carried out confocal time-lapse imaging to assess the cellular origin of tenocytes at different dorsal-ventral positions along the “V” of MTJ. Cells were tracked at high temporal resolution using the *nkx3*.*1*^*NTR-mCherry*^ transgenic reporter from 25 to 50 hpf. Tenocytes were identified at the end of the time-lapse movie, based on their position along the medial edge of the MTJ and the presence of cellular processes extending into the intersomitic space. By retrospective cell tracing, we showed that tenocytes can originate from progenitor cells at the dorsal sclerotome, ventral sclerotome, and sclerotome derived notochord associated cells (Fig 6A and S5 Video). Tenocytes along a given MTJ were almost exclusively generated by the immediate anterior somite (232/233 cells). Cells originating from either the dorsal or ventral sclerotome domains typically divided 1–2 times before generating 1–2 tenocytes along with other interstitial cells, likely osteoblast precursors. By contrast, sclerotome derived notochord associated cells often divided 2–3 times generating descendants including 1–4 tenocytes. For example, a single cell near the notochord (indicated by the green arrow in Fig 6A) underwent multiple cell divisions, generating 2 tenocytes at the dorsal MTJ and 4 interstitial cells occupying the space in between the notochord, muscles and the spinal cord. At 12 hours of imaging (37 hpf), we saw the first cell occupying the MTJ. After about 17.5 hours of time lapse imaging (41.5 hpf), we observed that cells had occupied and filled the “V” of MTJ, consistent with the emergence of *scxa* expression by 42 hpf (S6 Fig). Our result suggests that tenocytes in the zebrafish trunk are established by 42 hpf and are derived from both dorsal and ventral sclerotome domains as well as sclerotome derived notochord associated cells. Interestingly, we found that some cells in the dorsal sclerotome domain migrated dorsally and generated fin mesenchymal cells in the fin fold (Fig 6 and S5 Video), suggesting that the dorsal sclerotome also contributes to the formation of other dorsal appendages.

To better quantify the cell tracing data, we subdivided axial tenocytes into three different domains based on their final dorsal-ventral positions along the MTJ (Fig 6B). From the lateral view of the zebrafish, the “MTJ tip” represents the region of MTJ that sits directly on top of the notochord, while “dorsal MTJ” and “ventral MTJ” represents the regions of MTJ that are dorsal or ventral of the MTJ tip, respectively. We individually traced 233 tenocytes and correlated their final positions with the origin of progenitor cells (dorsal sclerotome, ventral sclerotome, and sclerotome derived notochord associated cells). Of the 233 tenocytes traced, 40 originated from the dorsal sclerotome, 108 from the ventral sclerotome, and 85 from sclerotome derived notochord associated cells (Fig 6C). The dorsal sclerotome mostly contributed to tenocytes along the dorsal MTJ, accounting for 97.5% of dorsal tenocytes traced (39/40 cells). However, we did find 1 tenocyte (2.5%) in the MTJ tip, suggesting that the dorsal sclerotome also has the capacity to generate tenocytes more ventrally along the MTJ. By contrast, the ventral sclerotome generated tenocytes predominantly in the ventral MTJ (29/108 cells, 26.9%) and MTJ tip (78/108 cells, 72.2%), and occasionally in the dorsal MTJ (1/108 cells, 0.9%). Sclerotome derived notochord associated cells generated tenocytes in the MTJ tip (21/85 cells, 24.7%) and dorsal MTJ (64/85 cells, 75.3%), but never tenocytes in the ventral MTJ. Since we previously showed that sclerotome derived notochord associated cells originate from the ventral sclerotome (Fig 1D and 1E), we conclude that the ventral sclerotome contributes to tenocytes in all 3 regions along the MTJ (Fig 6C). Together, our results suggest that the sclerotome of a given somite contributes to tenocytes along the posterior boundary of the same somite with a distinct spatial pattern: tenocytes along much of the MTJ come from the ventral sclerotome, while the dorsal sclerotome contributes to tenocytes primarily at the dorsal MTJ region.

### Loss of tenocytes compromises muscle integrity

**Fig 7:**
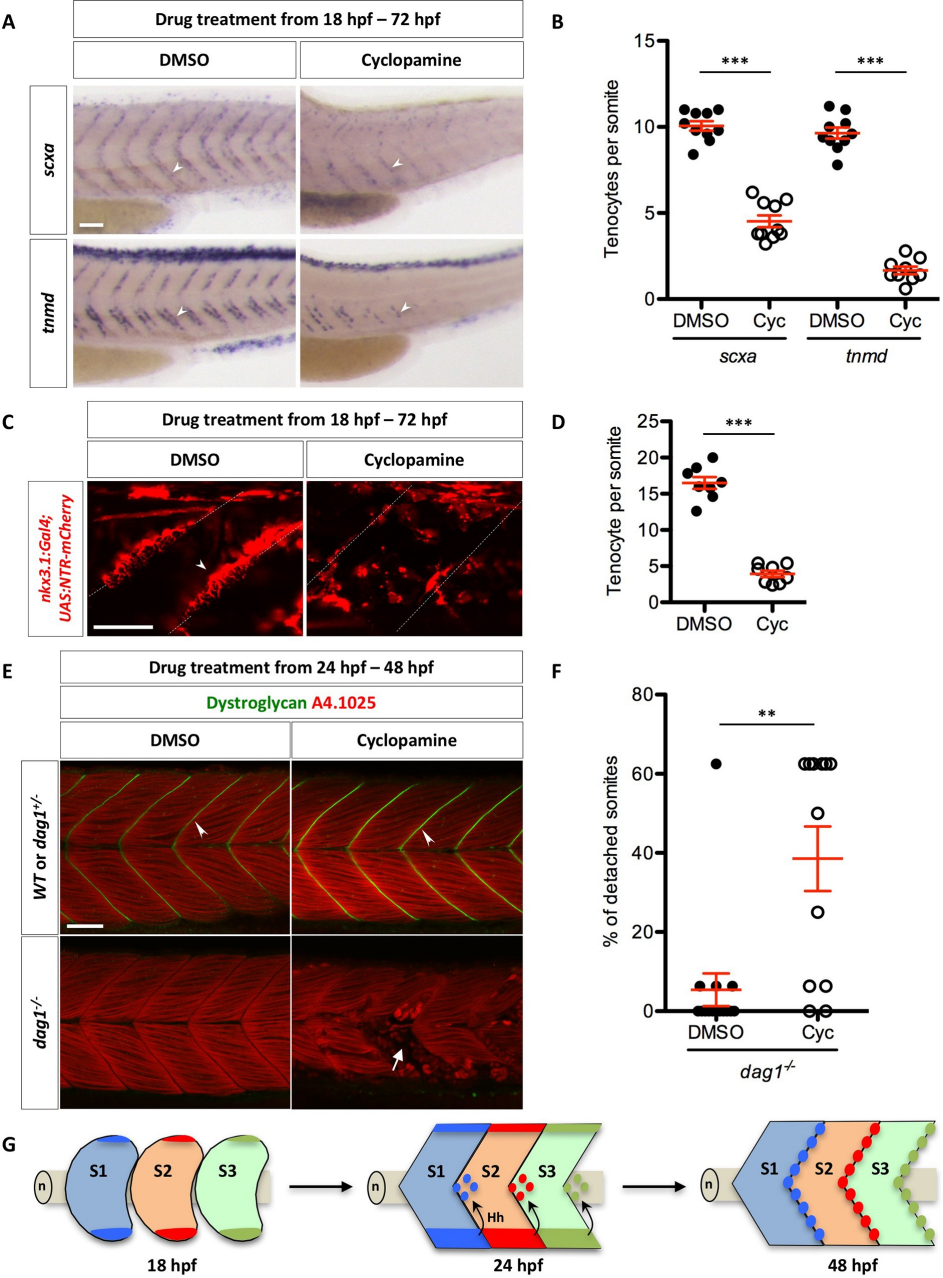
Regulation of tenocytes by Hh signaling. (A) Wild-type embryos were treated with DMSO or cyclopamine between 18 hpf and 72 hpf, and stained for the expression of *scxa* and *tnmd*. Expression of both *scxa* and *tnmd* in tenocytes (arrowheads) were reduced in the presence of cyclopamine compared to DMSO controls. *n* = 60 embryos per condition. (B) Quantification of the drug experiment in (A). The numbers of *scxa*^*+*^ and *tnmd*^*+*^ tenocytes along each MTJ were scored and averaged between somite 18 and somite 23 in DMSO/cyclopamine treated embryos. Each data point represents the average number from an individual embryo. Cyclopamine-treated embryos had significantly reduced numbers of *scxa*^*+*^ and *tnmd*^*+*^ tenocytes. *n* = 10 (DMSO/*scxa*, Cyc/*scxa* and DMSO/*tnmd*) and 9 (Cyc/*tnmd*) embryos. (C) *nkx3*.*1*^*NTR-mCherry*^ embryos were treated with DMSO or cyclopamine between 18 hpf and 72 hpf, and imaged to visualize tenocytes. Reduced number of tenocytes (arrowhead) along the MTJ (dotted lines) was observed in cyclopamine-treated embryos. *n* = 20 embryos. (D) Quantification of the drug experiment in (C). The number of tenocytes was quantified similarly as in (B). Cyclopamine-treated embryos had significantly reduced numbers of *nkx3*.*1*^*NTR-mCherry*^ tenocytes. *n* = 8 embryos per condition. (E) Embryos from a cross between two heterozygous *dystroglycan* fish (*dag1*^*+/-*^) were treated with either DMSO or cyclopamine from 24 hpf to 48 hpf, and stained for Dystroglycan (green) and myosin heavy chain (A4.1025, red). Dystroglycan was present along the MTJ (arrowheads) in sibling controls but absent in *dag1*^*-/-*^ fish. Most DMSO-treated *dag1*^*-/-*^ fish had normal muscle attachments despite the loss of Dystroglycan. By contrast, cyclopamine-treated *dag1*^*-/-*^ embryos had extensive muscle detachment as evident by the presence of coiled-up fibers (arrow). (F) Quantification of muscle detachment in drug treated *dag1*^*-/-*^ embryos shown in (E). *dag1*^*-/-*^ fish treated with cyclopamine showed a significant increase in the percentage of somites with detached muscles. *n* = 15 (DMSO) and 12 (Cyc) embryos. Scale bars: 50 μm. (B, D, F) All data are plotted with mean ± SEM indicated. Statistics: Mann-Whitney *U* test. Asterisks representation: p-value < 0.01 (**) and p-value < 0.001 (***). (G) Model of axial tenocyte formation in zebrafish. At 18 hpf, the dorsal and ventral domains of the sclerotome are induced independent of Hh signaling. By 24 hpf, cells begin to migrate from the ventral sclerotome and generate a population of cells surrounding the notochord (n). Cells always exit from the posterior end of the ventral somite, and eventually occupy an area around the notochord about one half-somite more posterior to the somite of origin. The migration of sclerotome derived notochord associated cells is dependent on Hh signaling. Hh signaling is subsequently required to maintain sclerotomal marker expression in these cells. By 48 hpf, the MTJ is populated by tenocytes derived from the sclerotome from the anterior somite. Three somites (S1, S2, and S3) corresponding to somite 17–19 are depicted in the model.

Since axial tenocytes originate from the sclerotome and the migration of sclerotome derived notochord associated cell is regulated by Hh signaling, we hypothesized that inhibition of Hh signaling would result in loss of tenocytes. To test this, we treated embryos with DMSO or cyclopamine from 18 hpf to 72 hpf, and stained fish with the tenocyte markers *scxa* and *tnmd* (Fig 7A and 7B). Cyclopamine-treated embryos showed significantly reduced number of *scxa*^*+*^ or *tnmd*^*+*^ tenocytes compared to control embryos. Similarly, cyclopamine-treated *nkx3*.*1*^*NTR-mCherry*^ embryos displayed about four-fold reduction in the number of tenocytes along the MTJ (Fig 7C and 7D). Interestingly, the few cells found along the MTJ in cyclopamine-treated *nkx3*.*1*^*NTR-mCherry*^ embryos did not have the same morphology as wild-type tenocytes (Fig 7C), suggesting that Hh signaling might also regulate the cellular morphology of tenocytes.

Since the MTJ plays an essential role in maintaining muscle integrity, we hypothesized that the reduction in tenocytes along the MTJ may affect muscle attachment. To test this possibility, we utilized the *dystroglycan* (*dag1*) mutant, a well-established model of muscular dystrophy in zebrafish [30,31]. *dag1* homozygous mutants have normal early muscle development but develop a progressive muscle detachment phenotype starting at 2 dpf [30,31]. Wild-type embryos or *dag1* mutants were treated with DMSO or cyclopamine from 24 hpf to 48 hpf, and then stained with antibodies to label Dag1 protein and all myofibers (A4.1025) (Fig 7E and 7F). Cyclopamine-treated wild-type or *dag1*^*+/-*^ embryos showed normal Dag1 localization along the MTJ and normal muscle attachments as controls. By contrast, cyclopamine-treated *dag1*^*-/-*^ mutants displayed a substantial increase in muscle detachment compared to DMSO-treated controls. Thus, loss of tenocytes by the inhibition of Hh signaling correlates with significantly enhanced muscle detachment phenotype. Although we cannot rule out the possibility that cyclopamine might compromise muscle integrity independent of tenocytes, our results suggest that tenocytes play an important role in maintaining muscle integrity.

## Discussion

In this study, we characterize the development of the sclerotome in zebrafish and show that the sclerotome generates axial tenocytes. First, the zebrafish sclerotome consists of two separate domains in the somite, distinct from mouse and chick. Second, Hh signaling is not required for the induction of the sclerotome, but is essential for the migration and maintenance of sclerotome derived cells. Third, different domains of the sclerotome give rise to tenocytes in a stereotypic pattern, and loss of tenocytes results in enhanced susceptibility to muscle detachment.

### Identification of a novel dorsal sclerotome domain in zebrafish

Using classic sclerotome markers, we identified one domain located at the ventromedial region of each somite similar to what has been described in mouse and chick, suggesting that some aspects of sclerotome development are conserved across evolution. Interestingly, we also identified a smaller dorsal domain, located at the dorsomedial edge of the somite, which has not been previously described in any species. Four lines of evidence suggest that the dorsal domain is a true compartment of the sclerotome. First, the dorsal domain expresses key sclerotome markers identified in mouse and chick similar to the ventral domain, such as *nkx3*.*1*, *pax9*, *twist2* and *foxc1b*. Second, the dorsal domain is distinct from the myotome and the neural crest lineage. Third, the dorsal compartment is not derived from cells migrating from the ventral domain. By the time the earliest marker expression emerges in sclerotome derived cells sprouting from the ventral domain at 20 hpf, the dorsal and ventral sclerotome domains are already separate, suggesting that these two domains are induced independently. Most importantly, at the functional level, both the dorsal and ventral sclerotome domains give rise to tenocytes in a similar stereotypic manner. Together, our results suggest that zebrafish sclerotome consists of two separate domains, at the dorsomedial and ventromedial edge of each somite. Consistent with our findings, *twist1* has been shown to express in the dorsal region of each somite in medaka [25]. Similar dorsal expression has also been observed in *nkx3-1* in Xenopus (Xenbase). We propose that the bipartite sclerotome organization is conserved among lower vertebrates.

Why does zebrafish process two separate sclerotome domains rather than one single continuous domain as in mouse and chick? One possibility is that the dorsal sclerotome domain in zebrafish is required to generate some dorsal structures unique to lower vertebrates. Consistent with this idea, cell tracing in *nkx3*.*1*^*NTR-mCherry*^ transgenic fish showed that some cells from the dorsal sclerotome domain migrate dorsally and contribute to fin mesenchymal cells in the fin fold. Therefore, the dorsal sclerotome might be a unique feature for finned species. Since mice and chick do not have fins, the dorsal domain is not maintained and thus eliminated during evolution. An alternative scenario is that the difference in sclerotome organization between teleosts and higher vertebrates is due to the different requirements for bone and cartilage. In land animals, the skeletal structure must be able to resist the forces of gravity in addition to providing structural support for skeletal muscles. Because of the requirement for a more sophisticated skeleton, higher vertebrates such as mouse and chick might allocate a much larger proportion of the somite for the sclerotome to generate the required bone and cartilage. In contrast, zebrafish requires a larger myotome to generate the large muscles required for movement in their viscous environment. It is therefore plausible that during evolution, the increasing size of the sclerotome eventually merges the two separate sclerotome compartments, resulting in one large single sclerotome domain seen in higher vertebrates. To distinguish these two scenarios, one will need to perform detailed analysis of sclerotome development across different species along the evolutionary tree.

The divergence in sclerotome development between amniotes and teleosts is reminiscent of differential requirements of the notochord and the sclerotome in vertebral column formation. In amniotes, the segmentation of vertebral columns is determined by the sclerotome while the notochord is not intrinsically segmented [52]. By contrast, recent work has demonstrated that segmentation of axial skeleton in zebrafish is dictated by notochord sheath cells but not the somite segmentation clock [53–56]. Combining our results, it suggests that the divergence in sclerotome development between amniotes and teleosts might underlie their different roles in vertebral column segmentation.

### Unique roles of Hh signaling in sclerotome development

Hh signaling is an essential regulator of sclerotome development in vertebrates [37]. Early work in zebrafish shows that manipulation of Hh signaling results in changes in sclerotome marker expression [57]. Our work clarifies the distinct roles of Hh signaling during sclerotome development in zebrafish. In the absence of active Hh signaling, both the dorsal and ventral sclerotome domains still form normally. However, the compartment marked by sclerotome derived notochord associated cells is completely lost. Consistent with this finding, active Hh signaling, indicated by the expression of Hh pathway target gene *ptc2*, is only present in sclerotome derived cells around the notochord but is absent from the dorsal and ventral sclerotome domains. Together, these results suggest that Hh signaling is not required for the initial induction of sclerotome domains but is essential for the maintenance of sclerotome derived cells. Our model is in agreement with the phenotype of mouse *Shh* knock-out mutants, in which sclerotome formation still occurs, but the sclerotome appears to be much smaller with drastically reduced *Pax1* expression [10], suggesting a conserved role of Hh signaling in sclerotome development. Indeed, the induction of sclerotome is likely due to the inhibition of BMP signaling, as elimination of two BMP antagonists, Noggin (Nog) and Gremlin1 (Grem1), in *Nog;Grem1* double mutants, results in complete disruption of sclerotome initiation [58]. Whether BMP signaling plays a similar role in zebrafish sclerotome induction remains to be determined.

Combining cell tracing and marker analysis, our study reveals two distinct functions of Hh signaling during sclerotome development. First, Hh signaling is required for the directed migration of cells from the ventral sclerotome to surround the notochord. Photoconversion-based cell tracing experiments showed that cells fail to migrate out of the ventral sclerotome in embryos treated with a Smo antagonist, cyclopamine. Second, Hh signaling is required for the sustained marker expression in sclerotome derived notochord associated cells. Inhibition of Hh signaling after sclerotome derived cells finish their migration completely blocks the expression of sclerotome markers. As Shh is expressed in the notochord and the floor plate, it is conceivable that Shh first functions as a chemoattractant to mediate the migration of sclerotome derived cells towards the notochord, and then Shh activates the canonical Gli-dependent pathway in sclerotome derived cells to activate downstream target gene expression. Consistent with this model, previous work has shown that the transcriptional and chemotactic responses of Hh signaling bifurcate at or downstream of Smo, and cyclopamine can block Smo-dependent chemotaxis in commissural axons *in vivo* and cultured cells *in vitro* [59,60]. However, whether the migration of sclerotome cells is independent of Gli-mediated transcription still needs to be tested.

### Tenocytes originate from the sclerotome

Early work in chick embryos has shown that tenocytes are derived from a somitic compartment in between the myotome and sclerotome, termed the syndetome [18,19]. It is important to note that although it is described as a fourth somitic compartment, the syndetome is induced from *Pax1*^*+*^ sclerotome. Our analysis has revealed striking similarities in tenocyte development between zebrafish and higher vertebrates. First, zebrafish tenocytes are located between somite boundaries along the myotendinous junctions. This organization is conserved in mouse and chick, where tenocytes are found in the intersomitic mesenchyme between neighboring somites [22]. Second, zebrafish tenocytes have a similar profile of marker expression: they first express the early tenocyte progenitor marker *scxa*, and later express the differentiated tenocyte markers *tnmd* and *col1a2*. Third, by taking advantage of the perdurance of the mCherry protein in the *nkx3*.*1*^*NTR-mCherry*^ transgenic line, we show that both the dorsal and ventral sclerotome domains can give rise to tenocytes along the myotendinous junctions. Similar to what has been described in chick, by the time tenocytes begin to express *scxa* at 36 hpf, expression of sclerotome markers in these cells has largely disappeared. Together, our analysis shows that trunk tenocytes in zebrafish are analogous to axial tenocytes in higher vertebrate in their anatomical locations, molecular signature, and developmental origin. In agreement with our findings, gene expression studies suggest that myoseptal cells in trout show similar characteristics of tenocytes in zebrafish and likely arise from the sclerotome [61]. Thus, the generation of tenocytes from the sclerotome is an evolutionarily conserved process in vertebrates.

Our high-resolution analysis also reveals several novel features of tenocyte development in zebrafish. First, a mosaic labeling technique reveals that tenocytes display a unique morphology. Their cell bodies are located along the medial boundary of the myotendinous junction, and they project tree-like processes laterally into the intersomitic interface. These tenocyte processes are on average 40 μm in length, and can sometimes extend across the entire depth of the somite. As a result, the intersomitic space along the myotendinous junction is filled with fine tenocyte processes, consistent with previous ultrastructural studies of the MTJ by transmission electron microscopy [47]. This unique morphology might allow for maximal interactions between tenocytes and neighboring muscle fibers. Second, our cell tracing experiments show that different sclerotome domains make topologically distinct contributions to tenocytes. The dorsal domain generates primarily tenocytes at the dorsal MTJ, whereas the ventral domain can give rise to tenocytes along the entire dorsal-ventral axis. Cyclopamine-treated embryos show a severe reduction in the number of tenocytes, suggesting that Hh signaling also regulates tenocyte formation from the dorsal and ventral sclerotome domains. Third, our time-lapse movies show that the formation of tenocytes is a highly dynamic process, in which cells migrate out of the sclerotome, and undergo multiple rounds of cell divisions. Some of the daughter cells migrate to locate along the MTJ and change their multipolar mesenchymal morphology to tree-like tenocyte morphology, projecting long cellular processes into the space between the MTJ. This process is in contrast with the previous static view of tenocyte formation, in which tenocytes are induced at the boundary of the sclerotome by FGF (fibroblast growth factor) secreted from the myotome [18,19]. Lastly, previous chick-quail chimera experiments suggest that a given *Scx*-expressing domain between two somites arises from the same two neighboring somites [19]. By contrast, we show that sclerotome domains from a given somite exclusively give rise to tenocytes located at the posterior boundary of the same somite. In an extreme rare case (1/233), we found sclerotome cells from one somite gave rise to a tenocyte located at the posterior boundary of the next somite. Our result is in contrast with the “resegmentation” model in vertebral formation, where a single vertebral column is formed by sclerotomes from two neighboring somites [26,62]. In zebrafish, tenocytes along a given MTJ all arise from the same somite immediately anterior to the MTJ.

### Function of tenocytes in maintaining muscle integrity

Tendons are ECM-rich structures that connect muscles with bones. In zebrafish, the myotendinous junction is analogous to mammalian tendon, essential for the attachment of flanking muscle fibers. Interestingly, we show that axial tenocytes are located along the MTJ with their extensive processes sandwiched between adjacent somites. This raises the question: do zebrafish tenocytes play a regulatory role in maintaining muscle attachment? Based on the tenocyte morphology, it is plausible that ECM proteins, such as Col1a2, are synthesized in tenocyte cell bodies at the medial boundary of the MTJ, transported via cellular projections, and secreted in the extracellular space along the entire MTJ. In support of this idea, compromising tenocyte formation by the inhibition of Hh signaling results in a severely augmented muscle detachment phenotype in *dystroglycan* mutants. Similarly, depletion of Thrombospondin 4b (Tsp4b), an ECM protein secreted by putative tenocytes, in zebrafish, results in muscle detachments upon contraction due to defects of ECM assembly in the MTJ [46]. Although the precise mechanism of how tenocytes regulate muscle attachment is likely to be more complicated, our work highlights the importance of non-muscle cells in regulation of muscle integrity.

In summary, our study suggests the following steps for axial tenocytes formation in zebrafish (Fig 7G): (1) two distinct sclerotome domains in each somite are induced independent of Hh signaling. (2) Hh signaling promotes the migration of sclerotome derived cells to surround the notochord, and maintains the sclerotomal marker expression in these cells. (3) Both sclerotome domains and sclerotome derived notochord associated cells give rise to tenocytes along the MTJ in a stereotypic pattern. (4) Tenocytes undergo morphological changes to extend tree-like cellular processes into the intersomitic space. Thus, our work shows that axial tenocytes in zebrafish arise from bipartite sclerotome domains and are essential for maintaining muscle integrity.

## Materials and methods

### Ethics statement

All animal research was conducted in accordance with the principles outlined in the current Guidelines of the Canadian Council on Animal Care. All protocols were approved by the Animal Care Committee at the University of Calgary (#AC17-0128).

### Zebrafish strains

Zebrafish strains used in this study were maintained and raised under standard conditions. The following transgenic strains were used in this study: *ptc2*:*Kaede* [45], *col1a2*:*Gal4*, *col1a2*:*GFP*, *nkx3*.*1*:*Gal4*, *ola-twist1*:*EGFP*, *UAS*:*NTR-mCherry* [49], *UAS*:*Kaede* [49], and *α-actin*:*GFP* [63]. We also used the following mutant strains in this study: *igu*^*ts294*^ [44] and *dag1*^*hu3072*^ [31]. Mutant strains were maintained as heterozygotes and bred to generate homozygous mutants.

### Generation of transgenic lines

The *ola-twist1*:*EGFP* construct contains the 5 kb *twist1* promoter from medaka (*Oryzias latipes*) driving EGFP expression. The *ola-twist1*:*EGFP* transgenic line was generated by standard Tol2-mediated transgenesis. To generate *nkx3*.*1*:*Gal4* transgenic fish, BAC clone zC21G15 from the CHORI-211 library that contains the *nkx3*.*1* genomic region was used for bacteria-mediated homologous recombination following standard protocols [64]. zC21G15 contains 144 kb upstream and 45 kb downstream regulatory sequences of *nkx3*.*1*. First, an iTol2_amp cassette containing two Tol2 arms in opposite directions flanking an ampicillin resistance gene was recombined into the vector backbone of zC21G15. Next, a cassette containing the open reading frame of the transcriptional activator Gal4-VP16 with the kanamycin resistance gene was recombined into zC21G15-iTol2_amp to replace the first exon of the *nkx3*.*1* gene. After each round of recombination, successful recombinants were confirmed by PCR analysis. The final *nkx3*.*1*:*Gal4* BAC was then co-injected with *tol2* transposase mRNA into *UAS*:*NTR-mCherry* embryos at the one-cell stage. Positive transgenic lines were identified by screening mCherry expression in F1 embryos from injected founders. *col1a2*:*Gal4* and *col1a2*:*GFP* transgenic lines were generated similarly using BAC clone zC122K13 from the CHORI-211 library.

### In situ hybridization and immunohistochemistry

Whole-mount in situ hybridization and immunohistochemistry were performed according to previously established protocols. We used the following RNA probes in this study: *col1a2*, *crestin*, *foxc1b*, *mitfa*, *myoD*, *nkx3*.*1*, *nkx3*.*2*, *ntr-mCherry*, *pax1a*, *pax9*, *scxa*, *sox10*, *tnmd*, *twist1b*, *twist2*, and *versican b*. Double fluorescent in situ hybridizations were performed using different combinations of digoxigenin (DIG) or dinitrophenyl (DNP) labeled probes. For immunohistochemistry, the following primary antibodies were used: rabbit polyclonal antibody to Kaede (1:1000, MBL), mouse monoclonal antibody to Dystroglycan (1:20, Developmental Studies Hybridoma Bank (DSHB)), and mouse monoclonal antibody to myosin heavy chain (A4.1025, 1:20, DSHB). For fluorescent detection of antibody labeling, appropriate Alexa Fluor-conjugated secondary antibodies (1:500, Thermo Fisher) were used for this study.

### mRNA injections

Synthetic mRNAs were generated using the mMessage mMachine Kit (Ambion). Embryos at the one-cell stage were injected with 1 nL of mRNA solution to achieve the appropriate amount: 150 pg *Kaede* and 50 pg *tol2*. *Kaede*-injected embryos were screened at 18 hpf for homogeneous *Kaede* expression in somites to use in lineage tracing experiments.

### Kaede photoconversion

Photoconversion was carried out using the 405nm laser and the 20x objective on the Olympus FV1200 confocal microscope. At the appropriate stage, *Kaede*-expressing embryos were anesthetized using tricaine and mounted in 0.8% low melting point agarose. The duration and intensity of laser required for complete green-to-red conversion depended on the expression level of Kaede and the size of the target region. For cell tracing of the ventral sclerotome, typically a circular region of 20 by 20 pixels was converted using 1.5% 405nm laser for 4 seconds. For *ptc2*:*Kaede* photoconversion, a rectangular area of 1000 by 300 pixels was converted by scanning the area twice with 50% 405nm laser at 200 μs per pixel. After photoconversion, embryos were confirmed for expression of *Kaede*^*red*^ and recovered in E3 fish water until necessary stages.

### Time-lapse imaging and cell tracking

Time-lapse imaging of zebrafish embryos was carried out using the Olympus FV1200 confocal microscope. At the appropriate stage, fish were anesthetized using tricaine and mounted in 0.6% low melting point agarose. To keep the agarose hydrated and minimize the development of pigments as well as minimize movement of the fish, a small volume of E3 water with phenylthiourea and tricaine was carefully flooded around the agarose. To combine imaging with drug treatments, the drug was mixed at the appropriate concentration with the agarose prior to mounting. Z-stack images of the region of interest were then collected at regular intervals (5–10 mins) for up to 25 hours. Images were then compiled into movies using the Olympus Fluoview software. Cells from each movie were then manually tracked using Fiji [65].

### Vibratome sectioning

To obtain transverse views, in situ stained embryos were sectioned using a Leica VT1000S vibratome. Embryos post in situ hybridization were washed in PBS and mounted in a 20% gelatin solution. After solidifying, gelatin blocks were fixed overnight at 4°C. The gelatin blocks were then washed, trimmed, and mounted in the vibratome. Slices of 75 μm were sectioned from the zebrafish mid-trunk for imaging.

### Drug treatments

Embryos at the appropriate stage were treated in cyclopamine (Toronto Chemical) at a final concentration of 100 μM in E3 fish water. Control embryos were treated similarly in an equal concentration of DMSO. Treated embryos were grown to the desired stage for analysis. For cell ablation experiments, *NTR-mCherry*^*+*^ embryos and *NTR-mCherry*^*-*^ sibling controls at the desired stage were treated with metronidazole (MTZ, Sigma-Aldrich) at a final concentration of 5 mM in E3 fish water. Embryos were incubated in the MTZ solution for at least 24 hours and then fixed for analysis.

### Quantification of muscle detachment

Embryos from an incross of *dag1*^*+/-*^ fish were fixed at the appropriate stage and stained with Dystroglycan and myosin heavy chain (A4.1025) antibodies. *dag1*^*-/-*^ mutants were identified based on the absence of Dystroglycan staining along the MTJ. Stained embryos were imaged to visualize the integrity of myofibers between somite 2 and somite 17. The detachment percentage was calculated by dividing the total number of somites with detached fibers by the total number of somites scored (16).

## Supporting information

S1 Fig Characterization of the zebrafish sclerotome. (A) Double labeling of sclerotome markers in wild-type zebrafish at 24 hpf. *nkx3*.*1* and *pax9* expression overlaps in the dorsal sclerotome domain (short arrows), ventral sclerotome domain (long arrows), and sclerotome derived notochord associated cells (arrowheads). *pax1a* expression overlaps with *nkx3*.*2* (middle panel) and *nkx3*.*1* (bottom panel) only in the sclerotome derived notochord associated cells. The extent of the notochord is indicated by brackets. Non-specific labeling of notochord cells in *nkx3*.*2* staining is indicated by asterisks. *n* = 60 embryos per staining. (B) Expression of *twist1b*, *versican b*, and *foxc1b* in wild-type zebrafish at 24 hpf. All 3 markers are expressed in the dorsal sclerotome (short arrows), the ventral sclerotome (long arrows), and sclerotome derived notochord associated cells (arrowheads). *n* = 15 embryos per staining. (C) Time course analysis of sclerotome marker expression in wild-type zebrafish between 16 hpf and 22 hpf. Expression of *nkx3*.*1* and *pax9* begins to appear in the ventral sclerotome domain (long arrows) at 16 hpf and 18 hpf, respectively. By 18 hpf, *nkx3*.*1* is expressed in the dorsal sclerotome domain (short arrows). At 20 hpf, sclerotome derived cells (arrowheads) begin to “sprout” from the ventral domain, coinciding with the expression of *pax1a* and *nkx3*.*2*. Expression of *nkx3*.*1* and *pax9* is established in all three domains at this time. *twist2* is also expressed in the sclerotome and has a similar timing and expression pattern as *nkx3*.*1* and *pax9*. *n* = 30 embryos per staining. Scale bars: (A) 50 μm; (B, C) 200 μm.
(TIF)

S2 Fig Targeted labeling of the ventral somite by Kaede photoconversion. (A, B) Wild-type embryos injected with *Kaede* mRNA were photoconverted at 18 hpf in the ventral portion of one single somite, corresponding to the presumptive ventral sclerotome domain, as described in Fig 1C. Embryos (*n* = 9) were fixed and sectioned to examine the photoconverted region in cross-sections (A). Alternatively, embryos (*n* = 5) were remounted and imaged from the dorsal side. The resulting confocal stacks were 3D reconstructed to show transverse views of the photoconverted region (B). Strong *Kaede*^*red*^ signal (arrows) is restricted to the ventral portion of targeted somites (solid outlines), whereas deeper tissues are only weakly labeled (arrowheads). Note that a small patch of the skin (asterisks), corresponding to the point of laser entry, is also labeled by *Kaede*^*red*^. The neural tube (NT) and notochord (n) are indicated by dotted lines. (C) Corresponding color-coded depth projections of images shown in Fig 1D. At 24 hpf and 40 hpf, most *Kaede*^*red*^ cells (arrowheads) are found deeper in the fish compared to the photoconverted ventral somite. Deeper cells are indicated by red/magenta colors, while more superficial cells are represented by green/cyan colors. *n* = 35 embryos. Scale bars: 50 μm.
(TIF)

S3 Fig Characterization of the sclerotome. Wild-type embryos at 24 hpf were co-labeled with neural crest markers *crestin* (A, green), *mitfa* (B, green), or the muscle pioneer marker *eng2* (C, green), with *nkx3*.*1* (red). *nkx3*.1-expressing dorsal sclerotome (short arrows), ventral sclerotome (long arrows), and sclerotome derived notochord associated cells (arrowheads) do not express either *crestin*, *mitfa*, or *eng2*. The notochord (n) is indicated by brackets in lateral views and dotted lines in transverse views. *n* = 15 embryos per staining. Scale bars: 50 μm.
(TIF)

S4 Fig Sclerotome development in *iguana* (*igu*^*-/-*^) mutants. *igu*^*-/-*^ mutants and their sibling controls (*wt* or *igu*^*+/-*^) were stained with *nkx3*.*1*, *pax9*, *pax1a*, and *nkx3*.*2* at 24 hpf. In *wt* or *igu*^*+/-*^ controls, *nkx3*.*1* and *pax9* are expressed in the dorsal sclerotome domain (short arrows), the ventral sclerotome domain (long arrows), and sclerotome derived notochord associated cells (arrowheads), while *pax1a* and *nkx3*.*2* are expressed in sclerotome derived notochord associated cells only. In *igu*^*-/-*^ mutants, expression of all four sclerotome markers are absent or significantly reduced in sclerotome derived notochord associated cells, while expression of *nkx3*.*1* and *pax9* in the dorsal and ventral sclerotome domains remains unchanged. Images shown are lateral views with close-up views of boxed regions. Brackets indicate the location of the notochord. *n* = 30 embryos per staining. Scale bars: 200 μm.
(TIF)

S5 Fig Analysis of Hh response in the sclerotome. (A) *ptc2*:*Kaede* transgenic embryos were co-labeled using the *nkx3*.*1* probe (red) and the Kaede antibody (green) at 24 hpf. Neither the dorsal sclerotome domain (short arrows) nor the ventral sclerotome domain (long arrows) labeled by *nkx3*.*1* have overlapping expression with Kaede. *ptc2*:*Kaede* expression in slow muscle fibers are indicated by asterisks. *n* = 15 embryos. (B) *ptc2*:*Kaede* embryos were photoconverted at 24 hpf or 42 hpf, and imaged 6 hours later (top and bottom panel, respectively). *Kaede*^*green*^ signal represents “new” signaling activity within the 6-hour time window, whereas *Kaede*^*red*^ signal represents “old” signaling that occurs before the time of photoconversion. *ptc2*:*Kaede* expression is present in presumptive sclerotome derived notochord associated cells (arrowheads) at both 30 hpf and 48 hpf. *n* = 4 embryos per time point. Scale bars: 50 μm.
(TIF)

S6 Fig Time-course analysis of tenocyte marker expression in wild-type zebrafish. Expression of *scxa* and *tnmd* was analyzed every 6 hours between 24 hpf and 84 hpf. *scxa* expression appears in the ventral MTJ by 36 hpf and fills the entire “V” of the MTJ by 42 hpf. In contrast, *tnmd* expression appears at 42 hpf and expression remains restricted to the ventral portion of the MTJ until 60 hpf. From 60 hpf to 84 hpf, both *scxa* and *tnmd* expression are present in tenocytes along the entire “V” of the MTJ. Images at 72 hpf are also shown in Fig 4A. *n* = 15 embryos per staining. Scale bar: 200 μm.
(TIF)

S7 Fig Quantification of the length of tenocyte processes. *col1a2*^*Kaede*^ embryos at 5 dpf were imaged in transverse views and the length of tenocyte processes was measured for individual tenocytes. A representative image is shown in Fig 4D. Data is plotted with mean ± SEM indicated. *n* = 28 tenocytes from 15 embryos.
(TIF)

S1 Video Time-lapse imaging of migration of sclerotome derived cells in DMSO-treated embryos. Embryos injected with *Kaede* mRNA were photoconverted in the presumptive ventral sclerotome at 18 hpf, treated with DMSO at 22 hpf, and imaged over the next 20 hours. Sclerotome derived cells migrate from the ventral sclerotome to the notochord before dividing to generate a population of cells surrounding the notochord. Green arrows follow one representative sclerotome derived cell and its progeny. Snapshots of this video are shown in Fig 3. Scale bar: 50 μm.
(MP4)

S2 Video Time-lapse imaging of migration of sclerotome derived cells in cyclopamine-treated embryos. Embryos injected with *Kaede* mRNA were photoconverted in the presumptive ventral sclerotome at 18 hpf, treated with cyclopamine at 22 hpf, and imaged over the next 20 hours. Almost no sclerotome derived cells migrate towards the notochord. Snapshots of this video are shown in Fig 3. Scale bar: 50 μm.
(MP4)

S3 Video *nkx3*.*1*^*NTR-mCherry*^ reporter reveals tenocyte morphology. A confocal stack of lateral views of *nkx3*.*1*^*NTR-mCherry*^*; α-actin*:*GFP* embryos at 72 hpf were 3D rendered to visualize the tenocyte morphology within the MTJ. Video begins with the surface view of tenocytes (*nkx3*.*1*^*NTR-mCherry*^, red) with cellular projections seen extending through the MTJ between neighbouring somites (*α-actin*:*GFP*, green). The image is rotated 180 degrees along the horizontal axis to show the medial view where tenocyte cell bodies are found. The green channel is then removed and the image is rotated another 270 degrees to visualize tenocyte cellular projections along the MTJ. Scale bar: 50 μm.
(MP4)

S4 Video Time-lapse imaging of tenocyte formation in *nkx3*.*1*^*NTR-mCherry*^*; col1a2*:*GFP* embryos. *nkx3*.*1*^*NTR-mCherry*^*; col1a2*:*GFP* embryos were imaged at 26 hpf over 25 hours. One cell from the dorsal sclerotome (white arrow) and one cell from sclerotome derived notochord associated cells (cyan arrow) were traced. Their daughters are indicated by arrows of the same color. Solid arrows on the last frame indicates tenocytes. *mCherry*^*+*^ sclerotome derived cells are weakly positive for *col1a2*:*GFP* at 26 hpf. At the end of the movie, *mCherry*^*+*^ tenocytes show much stronger *col1a2*:*GFP* expression. Scale bar: 50 μm.
(MP4)

S5 Video Time-lapse imaging of tenocyte formation. *nkx3*.*1*^*NTR-mCherry*^ embryos were imaged at 25 hpf over 25 hours. Two cells from the dorsal sclerotome (white arrow and yellow arrowhead), one cell from sclerotome derived notochord associated cells (green arrow), and one cell from the ventral sclerotome (cyan arrow) were traced. Their daughters are indicated by arrows of the same color. Snapshots of this video are also shown in Fig 6. Scale bar: 50 μm.
(MP4)

S1 Table This table contains all the numerical data presented in this manuscript. (XLSX)
