# Supplementary figures and images for: Stereotypic generation of axial tenocytes from bipartite sclerotome domains in zebrafish
Source: PLoS Genet. 2018 Nov 2;14(11):e1007775. doi: 10.1371/journal.pgen.1007775 (PMC6235400; doi:10.1371/journal.pgen.1007775)

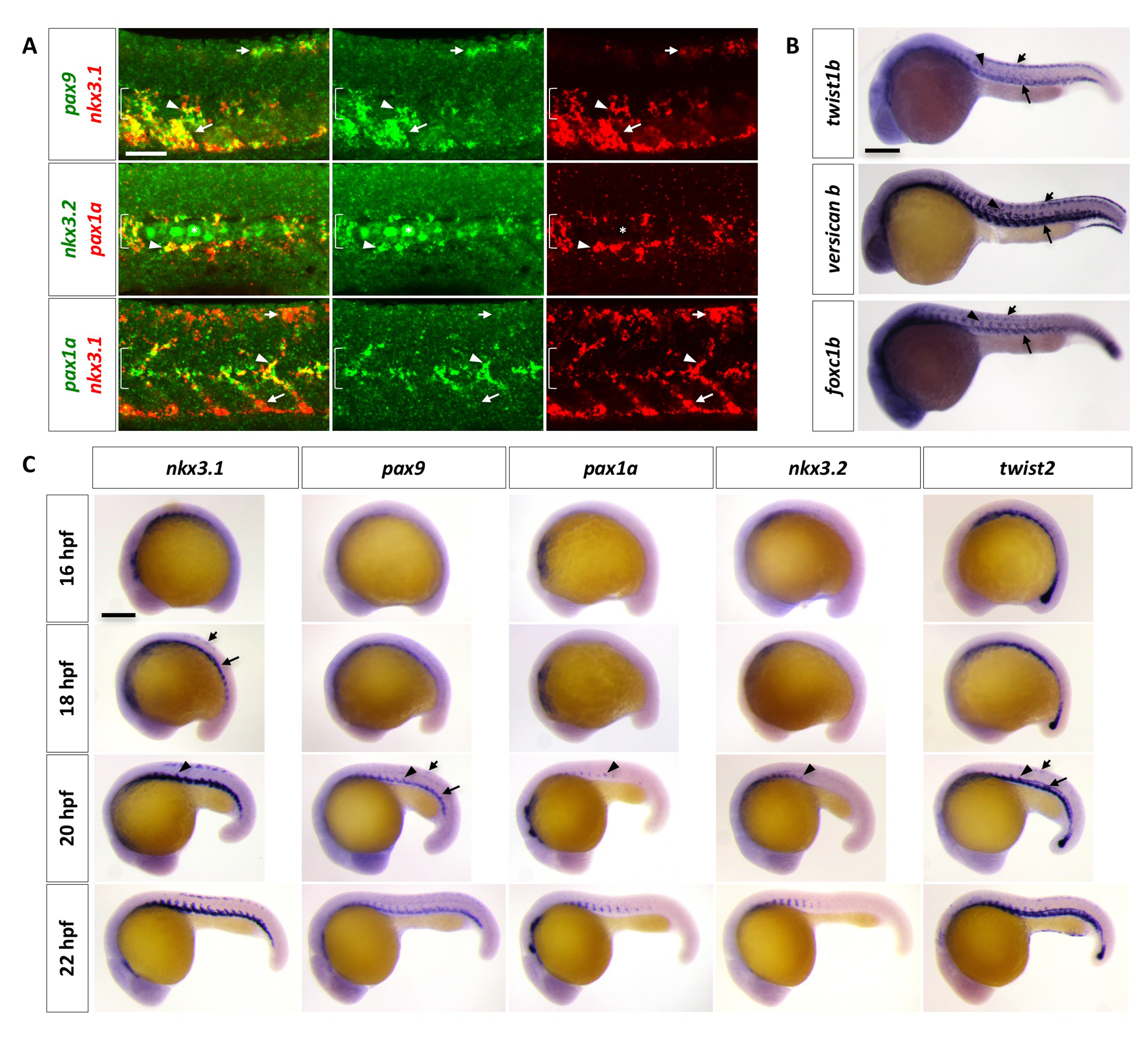

Supplement: S1 Fig — (A) Double labeling of sclerotome markers in wild-type zebrafish at 24 hpf. nkx3.1 and pax9 expression overlaps in the dorsal sclerotome domain (short arrows), ventral sclerotome domain (long arrows), and sclerotome derived notochord associated cells (arrowheads). pax1a expression overlaps with nkx3.2 (middle panel) and nkx3.1 (bottom panel) only in the sclerotome derived notochord associated cells. The extent of the notochord is indicated by brackets. Non-specific labeling of notochord cells in nkx3.2 staining is indicated by asterisks. n = 60 embryos per staining. (B) Expression of twist1b, versican b, and foxc1b in wild-type zebrafish at 24 hpf. All 3 markers are expressed in the dorsal sclerotome (short arrows), the ventral sclerotome (long arrows), and sclerotome derived notochord associated cells (arrowheads). n = 15 embryos per staining. (C) Time course analysis of sclerotome marker expression in wild-type zebrafish between 16 hpf and 22 hpf. Expression of nkx3.1 and pax9 begins to appear in the ventral sclerotome domain (long arrows) at 16 hpf and 18 hpf, respectively. By 18 hpf, nkx3.1 is expressed in the dorsal sclerotome domain (short arrows). At 20 hpf, sclerotome derived cells (arrowheads) begin to “sprout” from the ventral domain, coinciding with the expression of pax1a and nkx3.2. Expression of nkx3.1 and pax9 is established in all three domains at this time. twist2 is also expressed in the sclerotome and has a similar timing and expression pattern as nkx3.1 and pax9. n = 30 embryos per staining. Scale bars: (A) 50 μm; (B, C) 200 μm. (TIF) [file pgen.1007775.s001.tif]

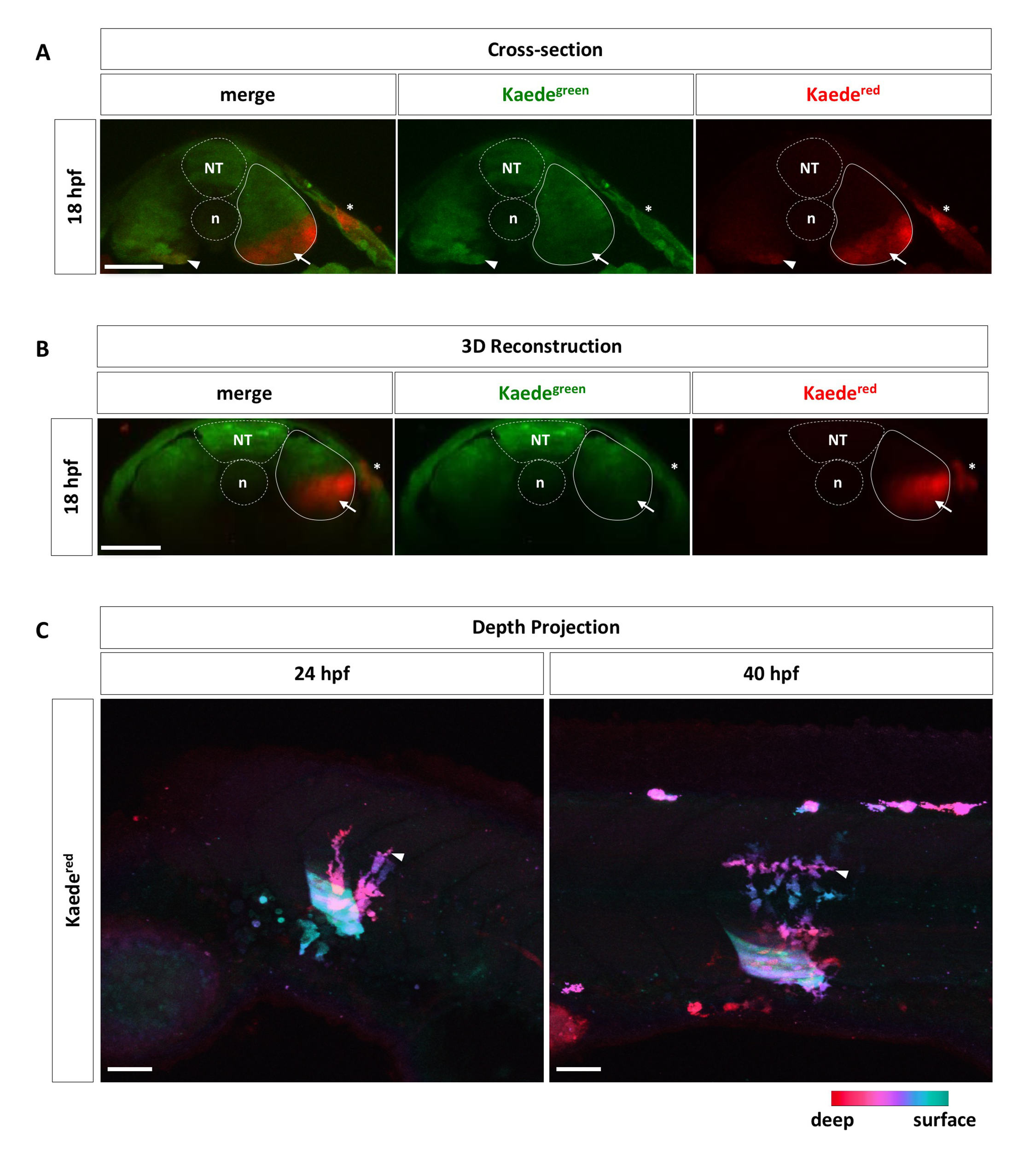

Supplement: S2 Fig — (A, B) Wild-type embryos injected with Kaede mRNA were photoconverted at 18 hpf in the ventral portion of one single somite, corresponding to the presumptive ventral sclerotome domain, as described in Fig 1C. Embryos (n = 9) were fixed and sectioned to examine the photoconverted region in cross-sections (A). Alternatively, embryos (n = 5) were remounted and imaged from the dorsal side. The resulting confocal stacks were 3D reconstructed to show transverse views of the photoconverted region (B). Strong Kaedered signal (arrows) is restricted to the ventral portion of targeted somites (solid outlines), whereas deeper tissues are only weakly labeled (arrowheads). Note that a small patch of the skin (asterisks), corresponding to the point of laser entry, is also labeled by Kaedered. The neural tube (NT) and notochord (n) are indicated by dotted lines. (C) Corresponding color-coded depth projections of images shown in Fig 1D. At 24 hpf and 40 hpf, most Kaedered cells (arrowheads) are found deeper in the fish compared to the photoconverted ventral somite. Deeper cells are indicated by red/magenta colors, while more superficial cells are represented by green/cyan colors. n = 35 embryos. Scale bars: 50 μm. (TIF) [file pgen.1007775.s002.tif]

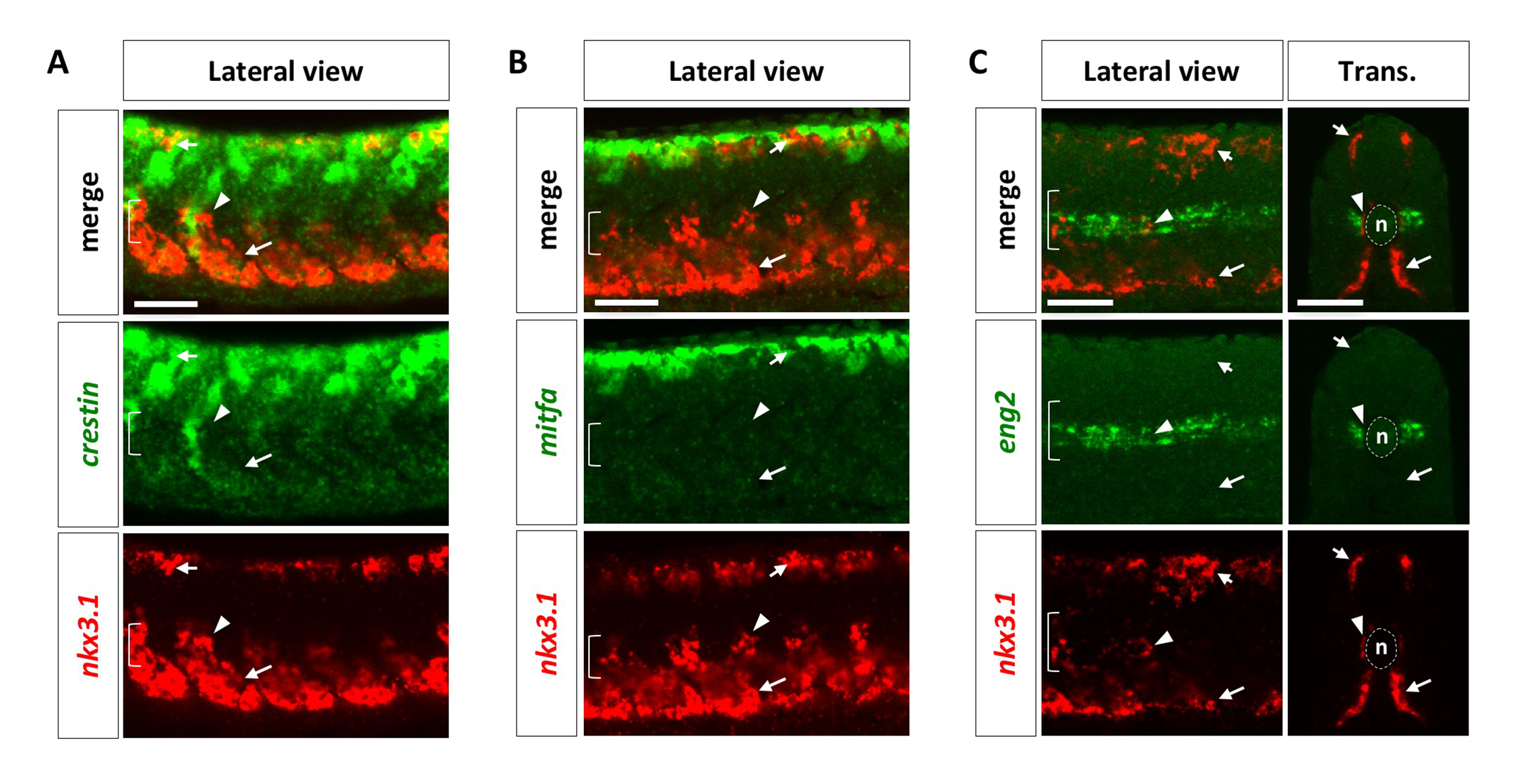

Supplement: S3 Fig — Wild-type embryos at 24 hpf were co-labeled with neural crest markers crestin (A, green), mitfa (B, green), or the muscle pioneer marker eng2 (C, green), with nkx3.1 (red). nkx3.1-expressing dorsal sclerotome (short arrows), ventral sclerotome (long arrows), and sclerotome derived notochord associated cells (arrowheads) do not express either crestin, mitfa, or eng2. The notochord (n) is indicated by brackets in lateral views and dotted lines in transverse views. n = 15 embryos per staining. Scale bars: 50 μm. (TIF) [file pgen.1007775.s003.tif]

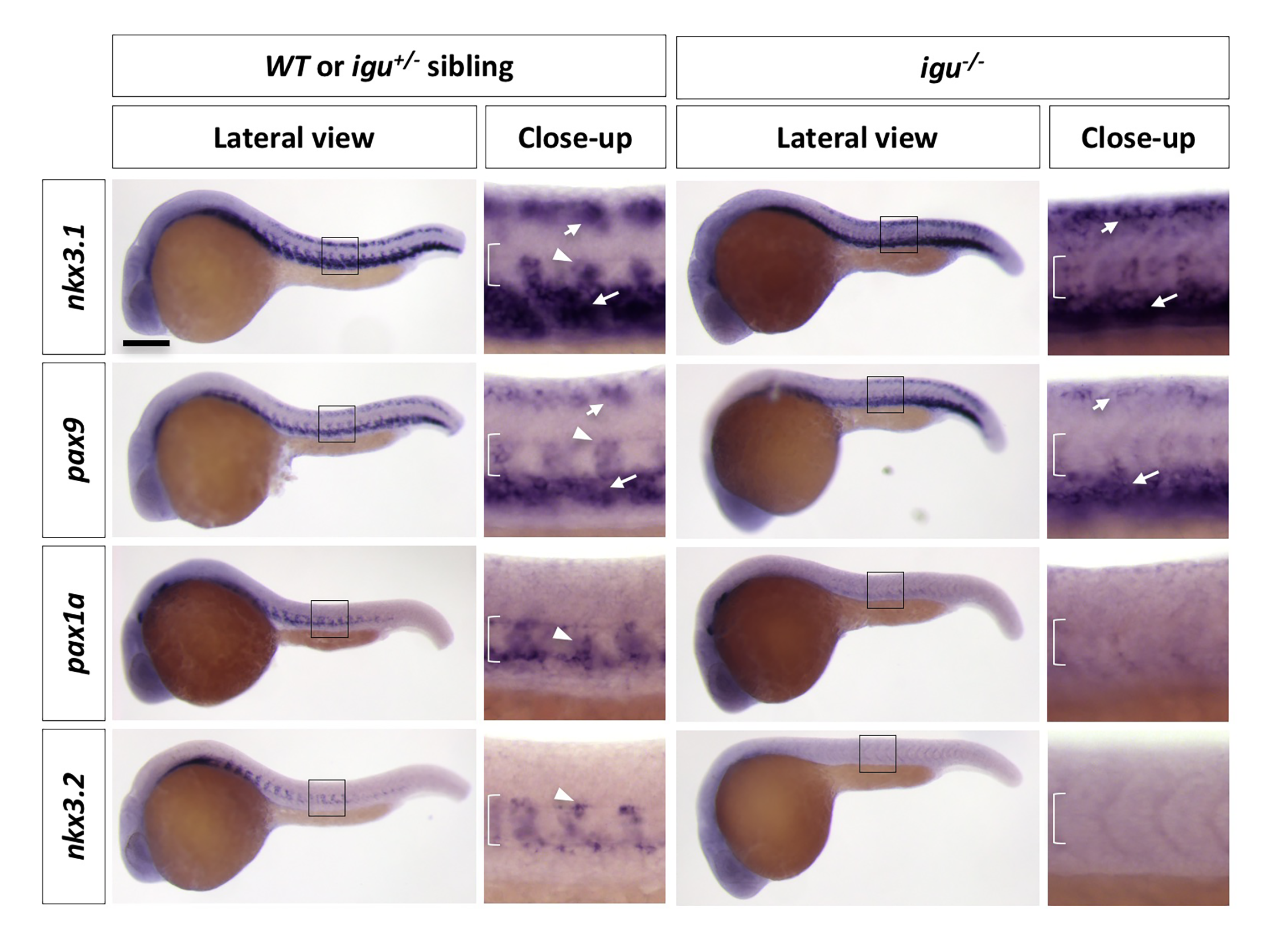

Supplement: S4 Fig — igu-/- mutants and their sibling controls (wt or igu+/-) were stained with nkx3.1, pax9, pax1a, and nkx3.2 at 24 hpf. In wt or igu+/- controls, nkx3.1 and pax9 are expressed in the dorsal sclerotome domain (short arrows), the ventral sclerotome domain (long arrows), and sclerotome derived notochord associated cells (arrowheads), while pax1a and nkx3.2 are expressed in sclerotome derived notochord associated cells only. In igu-/- mutants, expression of all four sclerotome markers are absent or significantly reduced in sclerotome derived notochord associated cells, while expression of nkx3.1 and pax9 in the dorsal and ventral sclerotome domains remains unchanged. Images shown are lateral views with close-up views of boxed regions. Brackets indicate the location of the notochord. n = 30 embryos per staining. Scale bars: 200 μm. (TIF) [file pgen.1007775.s004.tif]

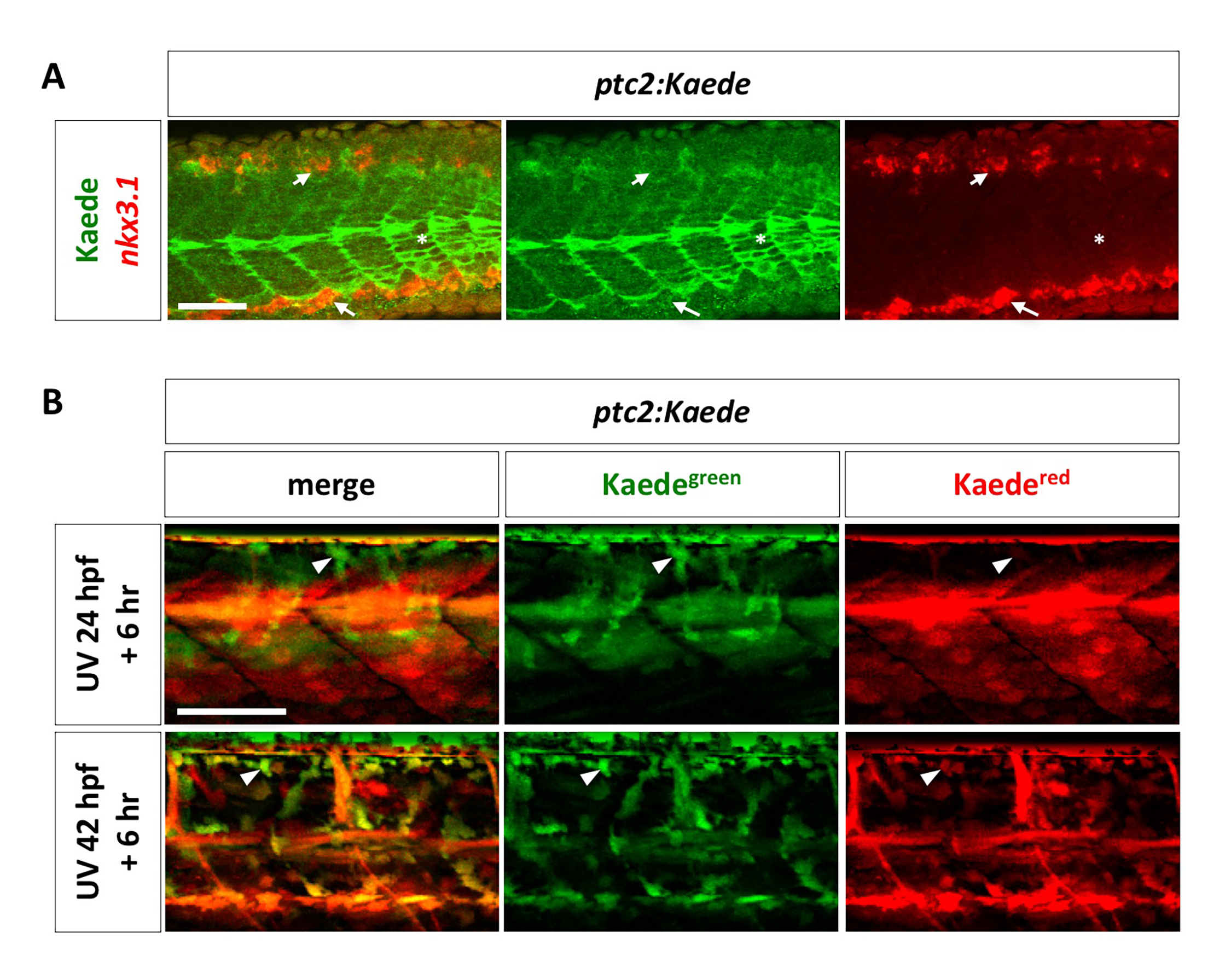

Supplement: S5 Fig — (A) ptc2:Kaede transgenic embryos were co-labeled using the nkx3.1 probe (red) and the Kaede antibody (green) at 24 hpf. Neither the dorsal sclerotome domain (short arrows) nor the ventral sclerotome domain (long arrows) labeled by nkx3.1 have overlapping expression with Kaede. ptc2:Kaede expression in slow muscle fibers are indicated by asterisks. n = 15 embryos. (B) ptc2:Kaede embryos were photoconverted at 24 hpf or 42 hpf, and imaged 6 hours later (top and bottom panel, respectively). Kaedegreen signal represents “new” signaling activity within the 6-hour time window, whereas Kaedered signal represents “old” signaling that occurs before the time of photoconversion. ptc2:Kaede expression is present in presumptive sclerotome derived notochord associated cells (arrowheads) at both 30 hpf and 48 hpf. n = 4 embryos per time point. Scale bars: 50 μm. (TIF) [file pgen.1007775.s005.tif]

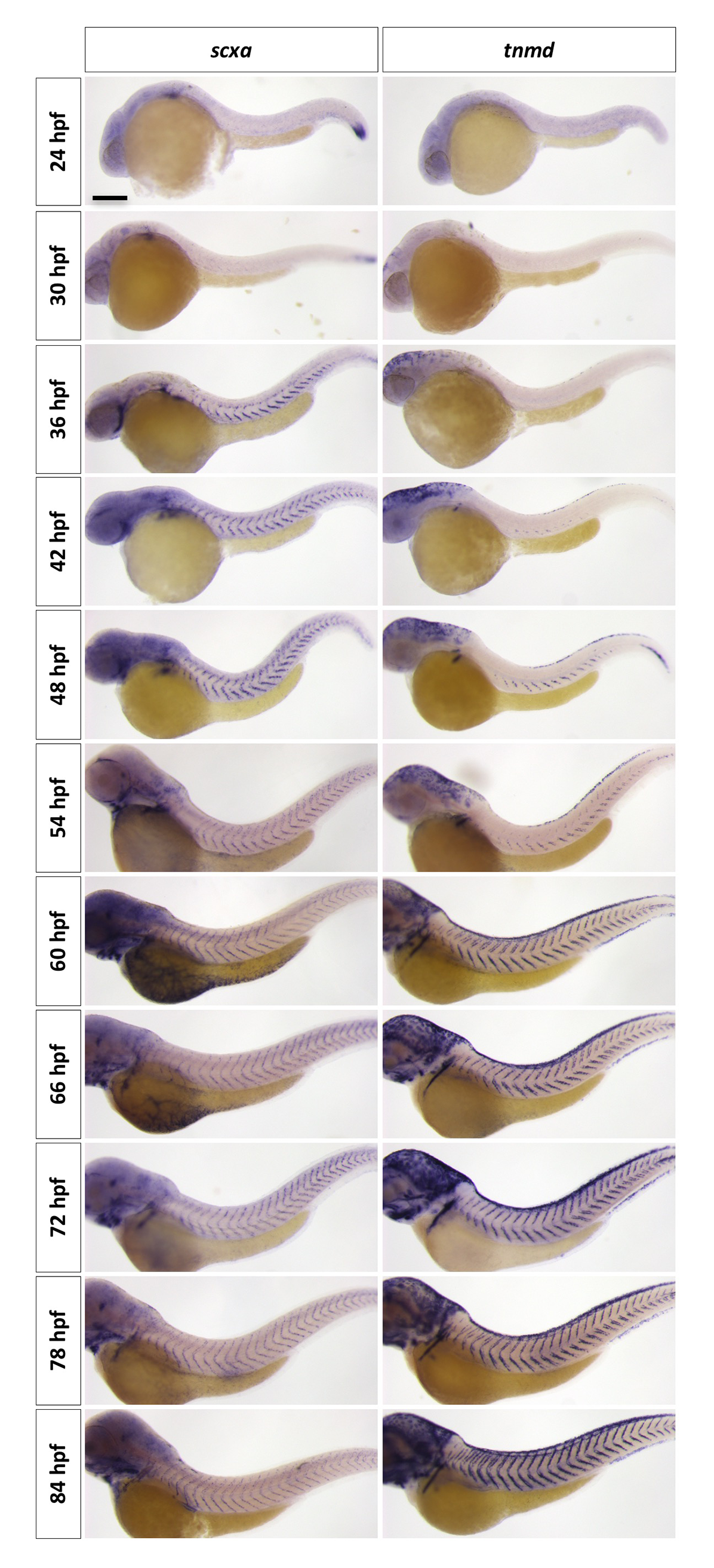

Supplement: S6 Fig — Expression of scxa and tnmd was analyzed every 6 hours between 24 hpf and 84 hpf. scxa expression appears in the ventral MTJ by 36 hpf and fills the entire “V” of the MTJ by 42 hpf. In contrast, tnmd expression appears at 42 hpf and expression remains restricted to the ventral portion of the MTJ until 60 hpf. From 60 hpf to 84 hpf, both scxa and tnmd expression are present in tenocytes along the entire “V” of the MTJ. Images at 72 hpf are also shown in Fig 4A. n = 15 embryos per staining. Scale bar: 200 μm. (TIF) [file pgen.1007775.s006.tif]

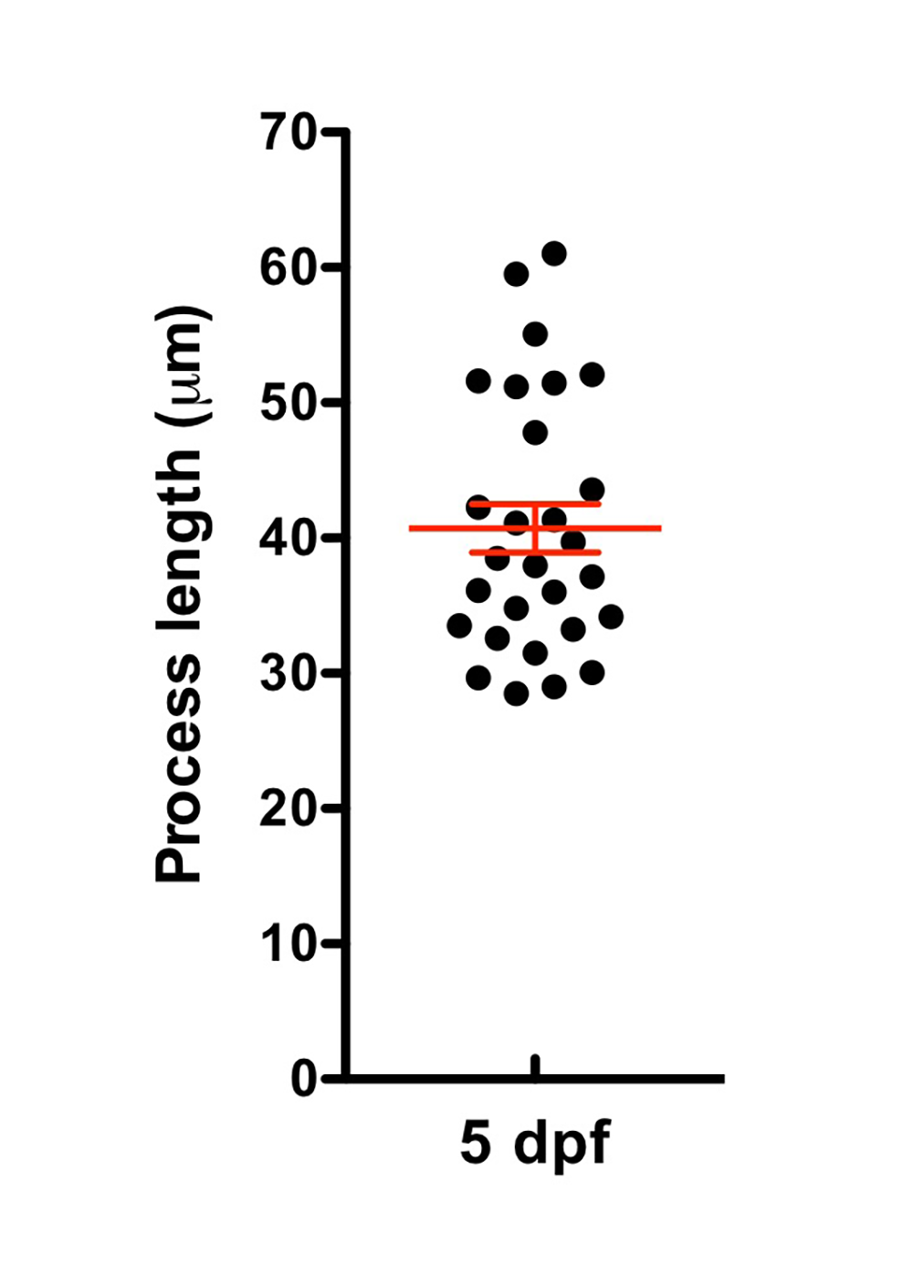

Supplement: S7 Fig — col1a2Kaede embryos at 5 dpf were imaged in transverse views and the length of tenocyte processes was measured for individual tenocytes. A representative image is shown in Fig 4D. Data is plotted with mean ± SEM indicated. n = 28 tenocytes from 15 embryos. (TIF) [file pgen.1007775.s007.tif]
